# Visible Light Optical Coherence Tomography: Technology and Biomedical Applications

**DOI:** 10.3390/bioengineering12070770

**Published:** 2025-07-17

**Authors:** Songzhi Wu, Shuo Wang, Baihan Li, Zhao Wang

**Affiliations:** School of Electronic Science and Engineering, University of Electronic Science and Technology of China, Chengdu 611731, China

**Keywords:** visible light OCT, biomedical imaging, functional imaging

## Abstract

Compared to widely used near-infrared OCT (NIR-OCT) systems, visible light OCT (vis-OCT) is an emerging imaging modality that leverages visible light to achieve high-resolution, high-contrast imaging and enables detailed spectroscopic analysis of biological tissues. In this review, we provide an overview of the state-of-the-art technology development and biomedical applications of vis-OCT. We also discuss limitations and future perspectives for advancing vis-OCT.

## 1. Introduction

Optical Coherence Tomography (OCT) has evolved into a cornerstone for biomedical research and clinical diagnostics since its first demonstration in 1991 [1]. OCT employs broadband low-coherence light sources combined with coherent gating techniques to detect depth-resolved backscattered/backreflected signals. By integrating laser scanning capabilities, OCT enables three-dimensional reconstruction of tissue structures. Owing to its non-invasive nature and high-resolution capabilities, OCT has revolutionized ophthalmic diagnostics and has expanded into various clinical fields, such as cardiology [2,3], dermatology [4], neurology [5], dentistry [6], gastroenterology [7], etc. Beyond structural imaging, functional OCT variations, including polarization-sensitive OCT [8], Doppler OCT [9], and OCT angiography (OCTA) [10], OCT elastography [11] provides additional contrast based on birefringence, flow dynamics, motion contrast, and strain-induced displacement sensing, respectively. These approaches offer multidimensional insights critical for both basic science research and clinical applications.

Most current OCT systems operate in the near-infrared (NIR) range, taking advantage of reduced tissue scattering and absorption to achieve millimeter-scale penetration in biological tissues. Despite its widespread use, NIR-OCT has inherent limitations that constrain its resolution and functional imaging capabilities. The relatively long wavelengths used in the NIR spectrum (typically 800–1300 nm) limit axial resolution, reduce superficial imaging contrast, and offer limited sensitivity to important chromophores such as hemoglobin. Visible-light OCT (vis-OCT) has emerged to overcome these limitations, offering several unique key advantages as follows:

1. Superior resolution: Axial resolution is inversely proportional to the square of the center wavelength, while lateral resolution scales linearly with wavelength, indicating that vis-OCT can achieve significantly higher axial and lateral resolution than NIR-OCT;
2. Hemoglobin absorption properties: The strong absorption of hemoglobin in the visible light range (especially 500–600 nm) enables quantitative measurement of microvascular oxygen saturation, making it a hallmark application of vis-OCT;
3. Enhanced contrast in superficial tissues: Increased scattering at shorter wavelengths improves imaging contrast in shallow tissue structures such as retinal layers.

These advantages collectively position vis-OCT as a promising modality for high-resolution, contrast-enhanced, and functionally informative imaging tool. Since the first groundbreaking study demonstrated a 0.5 μm axial resolution in biological tissues by integrating femtosecond lasers with photonic crystal fiber technology [12], vis-OCT has continuously evolved with significant technical advancements. The aim of this paper is to provide a comprehensive review of the current vis-OCT technology and its biomedical applications. The structure of the rest of the paper is organized as follows: Section 2 offers a review of vis-OCT theories, encompassing theoretical principles, key parameters, system evolution, vis-OCT-based functional imaging methods, and technical challenges in practical implementation. Section 3 summarizes the latest advancements of vis-OCT in biomedical imaging. Finally in Section 4, we discuss the advantages, limitations, challenges, and future perspectives for advancing vis-OCT technology.

The literature search for this review was conducted in May 2025 using multiple electronic databases, including PubMed, Web of Science, ScienceDirect, and IEEE Xplore, to systematically identify studies relevant to vis-OCT. The search strategy utilized a combination of keywords such as “visible-light OCT”, “vis-OCT”, and “retinal imaging”, using boolean operators (“AND”, “OR”) to optimize retrieval. Additional relevant articles were identified through manual screening of the reference lists from key publications. Studies were included if they were published in English, reported original research or comprehensive system development involving vis-OCT, and focused on technical innovation or biomedical imaging applications. Research articles and key conference reports are included with no restrictions in publication date. Although this review adopts a narrative structure, the search and selection process was designed to ensure systematic coverage of the relevant technical literature.

A recent review [13] has provided a valuable overview of vis-OCT, including clinical applications, system implementation, and select functional imaging strategies. In contrast, the present review offers a complementary perspective with a stronger emphasis on theoretical foundations, broader functional imaging methods, systematic analysis of technical challenges, broader coverage of emerging applications beyond ophthalmology, and recent progress in image processing and artificial intelligence.

## 2. Theory and System Implementation of Vis-OCT

### 2.1. Basic Theory and Key Components of Vis-OCT

**Figure 1:**
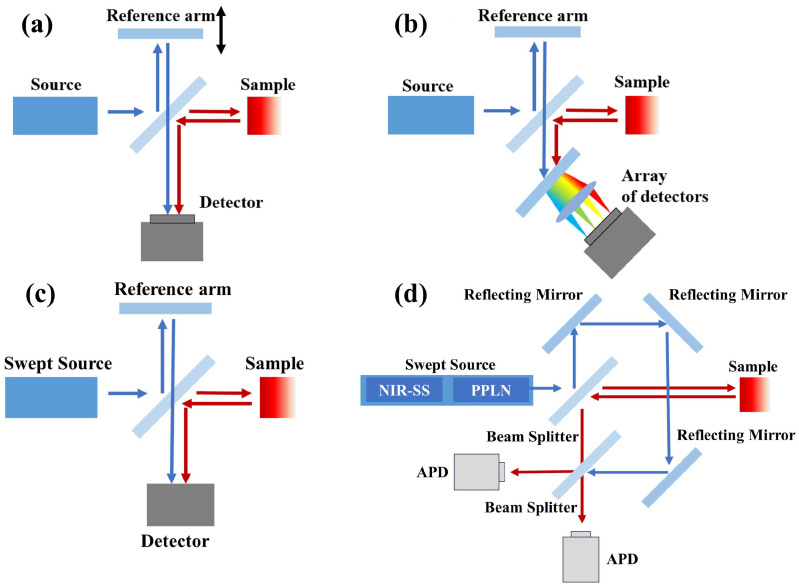
OCT system setups. (**a**) Early TD-OCT setup, (**b**) typical SD-OCT setup, (**c**) typical SS-OCT setup, and (**d**) visible light SS-OCT setup adopted in [16]. PPLN: fanout periodically poled lithium niobate; APD: avalanche photodetector.

OCT is based on low-coherence interferometry and has undergone significant technological evolution since its inception. As the earliest implementation, time-domain OCT (TD-OCT) laid the foundation for depth-resolved optical imaging by utilizing mechanically scanned optical delay line, as shown in Figure 1a. Early vis-OCT systems adopted TD setups [12,14]. However, TD-OCT suffers from the fundamental limitations of low imaging speed and low sensitivity due to the need to mechanically scan the reference arm, making it less suitable for high-throughput *in vivo* applications. To overcome these drawbacks, spectral-domain OCT (SD-OCT) has been developed, which retrieves depth-resolved backscattering/backreflection profiles by applying a Fourier transform to a spectral encoded interferogram, eliminating the need for mechanically scanning the optical delay line. In SD-OCT, the interference signal is spectrally dispersed by a diffraction grating and captured simultaneously by a linear detector array (Figure 1b), enabling faster acquisition rates, improved sensitivity, and enhanced system stability. SD-OCT has become the dominant configuration of vis-OCT today.

In addition to SD-OCT, another Fourier-domain configuration known as swept-source OCT (SS-OCT) has been developed [15], which replaces the broadband light source and spectrometer with a rapidly tunable long-coherence length laser and a single high-speed photodetector (Figure 1c). SS-OCT offers several additional advantages, including reduced sensitivity roll-off with depth, improved fringe detection efficiency, and higher imaging speeds due to its faster lasers and photodetectors as compared to spectrometers. However, in the context of visible-light OCT, SS-OCT has not yet seen widespread adoption due to the lack of mature swept lasers in the visible light range. A recent work [16] has presented an early SS-vis-OCT system (Figure 1d) using second-harmonic generation from NIR sources to overcome the sensitivity roll-off limitation of SD-OCT.

Below, we treat the basic theories of vis-OCT as in the form of SD-OCT. A typical vis-OCT system consists of four major components as follows: a light source, a reference and a sample arm, and a spectrum detection system. Physically, in SD-OCT, the detected interference arises from the superposition of the fields returning from the reference and sample arms, as shown in Figure 1b. The electric field from the light source can be expressed as follows [17]:

(1) $$E_{i}=s\left(k,\omega \right)e^{i(kz-\omega t)},$$

where *k* is the wavenumber, *z* is the position, and ω is the angular frequency. The backscattered/backreflected fields from the reference and sample arms, $E_{R}$ and $E_{S}$, with an optical path difference within the coherence length of the light source, interfere at the beamsplitter and are dispersed into different wavelengths (or wavenumbers) by a spectrometer and detected using a line scanning camera. The detected spectral intensity I(k) is as follows:

(2) I(k)=|ER(k)+ES(k)|2=|ER(k)|2+|ES(k)|2+2Re[ER∗(k)ES(k)],

The cross-correlation term 2Re[ER∗(k)ES(k)] contains the depth-resolved interference information. By acquiring the spectral interferogram I(k) and applying an inverse Fourier transform with respect to wavenumber *k*, the depth profile of sample reflectivity can be reconstructed without mechanically scanning the reference arm.

In SD-OCT systems, the axial and lateral resolution are decoupled and they define the ability of the OCT system to resolve the smallest structural features in different axes. The axial resolution can be expressed as follows:

(3) $$\Delta z=\frac{2\ln(2)}{\pi \cdot n}\cdot \frac{\lambda_{0}^{2}}{\Delta \lambda },$$

where *n* is the refractive index of the sample, $\lambda_{0}$ is the center wavelength of the light source, and Δλ is the bandwidth of the light source. The lateral resolution of OCT is typically formulated using Gaussian beam theory as follows:

(4) $$\Delta x=\frac{0.37\lambda_{0}}{NA},$$

This formula defines the idealized diffraction-limited lateral resolution, expressed as the full width at half maximum (FWHM) of the focal spot for a Gaussian beam. In practice, the actual lateral resolution is often degraded by additional factors such as optical aberrations, imperfect beam shaping, and system alignment errors.

### 2.2. Evolution of Vis-OCT Technology

This subsection reviews a series of significant advancements in vis-OCT, from its initial conceptualization to its current state. These developments have laid the foundation for both the theoretical and technical aspects of vis-OCT and have expanded its potential in diverse biomedical applications.

The concept of vis-OCT was first proposed by Povazay et al. in 2002 [12]. They utilized a sub-15-fs Ti:sapphire laser to pump a photonic crystal fiber, generating a shaped supercontinuum spectrum spanning 535–700 nm with a center wavelength near 600 nm. This broadband source enabled high-resolution OCT imaging in the visible range, achieving an axial resolution of approximately 0.6 μm in tissue. Moreover, this study recognized the wavelength-dependent absorption features of biological chromophores in the visible range, highlighting the potential of vis-OCT for both structural and functional imaging.

Parallel to this development, full-field OCT (FF-OCT) emerged as another important branch of visible light-based coherent imaging. Unlike scanning-based OCT systems, FF-OCT acquires *en face* tomographic images through parallel interferometric detection using image sensors such as charge-coupled device (CCD) or complementary metal-oxide-semiconductor (CMOS) cameras. In 2004, Dubois et al. [18] developed a white-light FF-OCT system operating in the visible range, achieving subcellular resolution using spatially incoherent halogen illumination and high-NA objectives. Subsequent advances such as dynamic FF-OCT (D-FF-OCT) and active phase modulation (APMD-FF-OCT) enabled the functional imaging of intracellular fluctuations and virtual histology [19,20]. While FF-OCT provides high-resolution *en face* imaging with minimal artifacts, its limited axial sectioning and functional capability make vis-OCT more suitable for depth-resolved and spectroscopic applications such as retinal oximetry and vascular imaging.

Despite its potential, early vis-OCT development was hampered by the lack of high-performance broadband visible light sources and slow acquisition speed. To address these challenges, Zhang et al. [21] developed a dual-band SD-OCT system that incorporated both visible and NIR wavelengths. By leveraging the fundamental and frequency-doubled outputs of a Ti:sapphire laser, the system enabled *in vivo* retinal imaging in rats. This design allowed for a direct comparison of retinal nerve fiber layer (RNFL) reflectivity between the two spectral bands, revealing distinct morphological features and demonstrating the utility of visible light for enhanced spectral contrast.

Building on growing interest in functional applications of vis-OCT, Yi et al. [22] conducted a landmark study demonstrating the first *in vivo* retinal oximetry using vis-OCT in pigmented rats. They constructed a free-space vis-OCT system and developed a comprehensive analytical model that incorporated light absorption, scattering, and red blood cell packing effects. By applying short-time Fourier transform (STFT) to extract depth-resolved spectra from the posterior wall of retinal vessels, they fitted the spectral data to their model to quantify hemoglobin oxygen saturation ($sO_{2}$). This approach enabled noninvasive measurement of mean arterial and venous $sO_{2}$ levels (95% and 72%, respectively), representing a critical step toward establishing vis-OCT as a quantitative three-dimensional functional imaging modality with broad applications.

**Figure 2:**
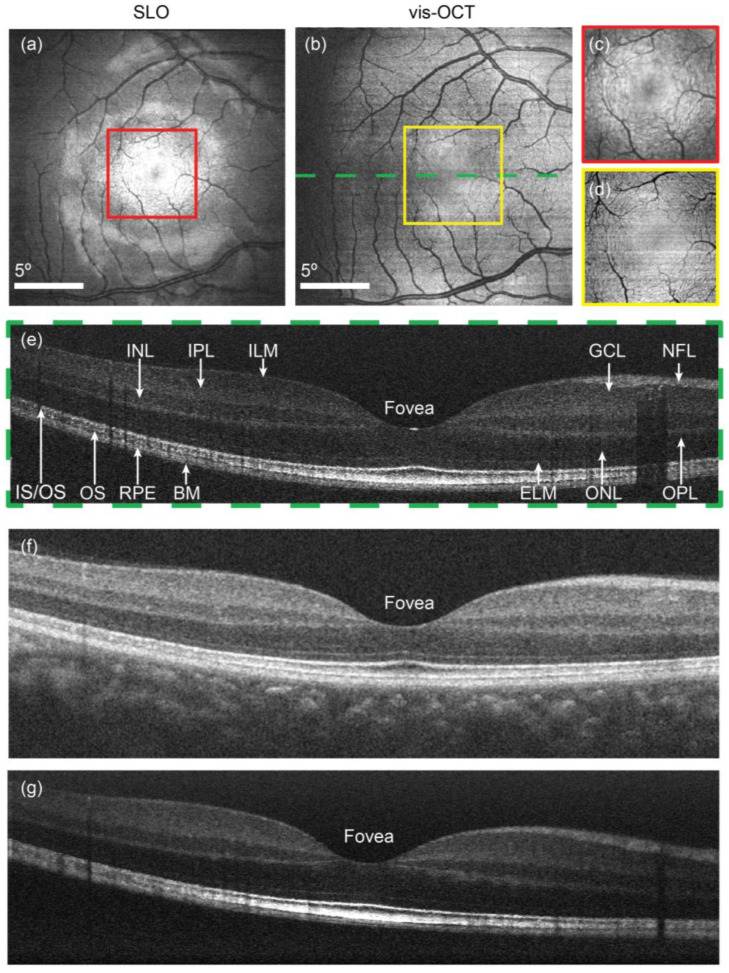
SLO and vis-OCT images centered at the fovea. (**a**,**b**) *En face* images of SLO and vis-OCT; (**c**,**d**) contrast-adjusted images from the squared area in (**a**) and (**b**); (**e**) cross-sectional vis-OCT image from the position highlighted in (**b**) with all anatomical structures labeled. ILM: inner-limiting membrane; NFL: neural fiber layer; GCL: ganglion cell layer; IPL: inner plexiform layer; INL: inner nuclear layer; OPL: outer plexiform layer; ONL: outer nuclear layer; IS/OS: inner/outer segment junction; OS: outer segment of photoreceptor; RPE: retinal pigmented epithelium; BM: Bruch’s membrane. (**f**) Single cross-sectional image using a commercial NIR-OCT system. (**g**) Averaged vis-OCT image from eight consecutive B-scans. The motion artifact was removed by aligning the adjacent B-scans. Reprinted with permission from [23] © Optical Society of America.

Extending their work to human subjects, Yi et al. [23] achieved the first *in vivo* retinal imaging in humans using vis-OCT, marking a significant milestone in the clinical translation. They developed a dual-modality system integrating vis-OCT with confocal scanning laser ophthalmoscopy (SLO) via a shared optical path, enabling co-registered cross-sectional and *en face* imaging. The system employed a supercontinuum light source centered at 564 nm with a 115 nm bandwidth, achieving an axial resolution of 0.97 μm in air. Compared to commercial NIR-OCT, vis-OCT offered enhanced contrast in the outer retina (e.g., photoreceptors and retinal pigment epithelium), despite with slightly reduced signals in the inner layers (Figure 2).

Later, Chen et al. [24] extended the vis-OCT’s functional capabilities by achieving comprehensive measurements of retinal $sO_{2}$ in healthy human subjects. They addressed the low SNR inherent to eye-safe imaging conditions by introducing a statistically grounded sampling method. This approach modeled OCT signal-to-noise using a Rice distribution, enabling the unbiased estimation of true OCT intensity. Spectral fitting of the interferometric signal to known absorption spectra of oxygenated and deoxygenated hemoglobin allowed for vessel-specific $sO_{2}$ quantification. The results revealed significant oxygenation differences between central retinal arteries and veins near the optic disc, as well as discernible variations across different arterial branches. Moreover, the statistical sampling strategy reduced $sO_{2}$ estimation error by approximately 3% compared to conventional averaging. This work marked a pivotal advance in translating vis-OCT from structural imaging to functional oximetry in humans, providing a critical foundation for future clinical applications in diseases involving retinal oxygen metabolism.

In 2020, Pi et al. [25] demonstrated the capability of vis-OCT to achieve cellular-level retinal imaging *in vivo*. By combining visible-light OCT with volumetric registration and averaging techniques, the authors significantly enhanced image resolution and contrast. This approach enabled clear visualization of fine retinal microstructures in live rats, including vitreous fibers, nerve fiber bundles, the vascular network, and sublaminar features within the inner plexiform layer (IPL). Additionally, individual ganglion cell bodies in the ganglion cell layer (GCL), nuclei in the inner nuclear layer (INL), as well as photoreceptor nuclei in the outer nuclear layer (ONL) and ellipsoid zone structures were successfully resolved. These results established vis-OCT as a powerful tool for noninvasively observing retinal architecture at the cellular scale, offering a promising avenue for identifying neuronal biomarkers and improving the early diagnosis of ophthalmic and neurodegenerative diseases.

Miller et al. [26] introduced vis-OCT fibergraphy as a non-invasive method for imaging retinal ganglion cell (RGC) axon bundles. Using a commercial small animal vis-OCT system, they achieved *in vivo* visualization of RGC axon networks in mice with 1.3 μm axial resolution. The study demonstrated that vis-OCT fibergraphy closely matched the ex vivo confocal microscopy results, with Sholl analysis confirming the quantitative agreement between both methods. This technique provided a detailed structural map of RGC axon bundles which could serve as a new biomarker for detecting early changes in optic neuropathies, offering improved diagnostics for conditions like glaucoma.

Conventional vis-OCT systems, despite offering high resolution and tissue contrast, have been hampered by limitations such as restricted field of view, reduced imaging depth, sensitivity roll-off, and shot-noise limit constraints. Addressing these challenges, Wang et al. [27] introduced a novel dual-channel vis-OCT system. Their innovative design features two key elements. First, to achieve shot-noise-limited performance, they implemented a per-A-line noise cancellation scheme. This technique uses a second, identical linear-in-k spectrometer to measure the source’s intensity noise directly from the reference arm, which is then digitally subtracted from the main interferogram to suppress excess noise. Second, to achieve full-range imaging, they employ reference pathlength modulation, which is accomplished by using a high-torque galvanometer to introduce a linear phase modulation during the scan. A subsequent Hilbert transform is applied to the data to reconstruct the full complex signal, which critically removes the complex–conjugate mirror image artifact and doubles the effective imaging depth. Consequently, the system delivers wide-field, full-range imaging, significantly improving imaging depth for clear visualization of both the retina and choroid. Furthermore, by substantially reducing noise sources through balanced detection, the system operates at shot-noise-limited performance, yielding high-quality images that surpass those of conventional vis-OCT.

In 2025, Fan et al. [16] introduced a swept-source vis-OCT system designed to mitigate the limitations of conventional spectral-domain vis-OCT, such as restricted imaging depth and sensitivity roll-off. Their approach involved generating a visible-light swept source via second-harmonic generation (SHG) from an amplified commercial NIR swept-source laser. This system demonstrated improved axial resolution (7.3 μm in air), significantly enhanced imaging depth (5 mm in air), and a 2.8-fold reduction in sensitivity roll-off. While this method advanced vis-OCT capabilities for research and potential clinical use by offering deeper penetration and better signal quality, challenges such as relatively lower output power (580 μW) and increased system complexity due to the SHG generation remain to be solved.

Here, we summarize the timeline of key vis-OCT technology development in Table 1.

**Table 1:** Key technological milestones of vis-OCT.

Year	Authors	Contributions
2002	Povazay et al. [12]	Proposed vis-OCT concept; achieved 0.6 μm resolution using a shaped visible supercontinuum light source.
2004	Dubois et al. [18]	Demonstrated white-light FF-OCT system with submicron axial resolution.
2011	Zhang et al. [21]	Developed the first dual-band OCT for *in vivo* rat retina imaging at visible and NIR wavelength range.
2013	Yi et al. [22]	Demonstrated the first *in vivo* retinal oximetry using vis-OCT and spectral modeling.
2015	Yi et al. [23]	Achieved the first human vis-OCT imaging with enhanced outer retina contrast.
2017	Chen et al. [24]	Enabled accurate human retinal $sO_{2}$ mapping via statistical noise modeling and spectral fitting.
2020	Pi et al. [25]	Achieved sub-cellular of retinal microstructures *in vivo*.
2020	Miller et al. [26]	Introduced vis-OCT fibergraphy for RGC axon imaging.
2024	Wang et al. [27]	Built a dual-channel vis-OCT with full-range, wide-field, shot-noise-limited imaging capability.
2025	Fan et al. [16]	Developed swept source-based vis-OCT with deeper imaging depth and reduced sensitivity roll-off using a SHG-based swept laser.

### 2.3. Vis-OCT Related Functional Imaging Methods

#### 2.3.1. Doppler Flow Measurement

Doppler OCT (DOCT) stands as an early and independent functional extension of OCT with its core purpose being the quantitative measurement of blood flow within tissues [28]. In recent years, DOCT has been incorporated into vis-OCT systems, where its role shifts from primarily improving blood flow quantification beyond what NIR-DOCT offers to enabling synergistic integration with vis-OCT’s intrinsic oximetric capabilities [29,30,31,32]. This combination facilitates the comprehensive assessment of crucial physiological parameters such as oxygen metabolism. While this synergistic approach is valuable, it encounters challenges because vis-OCT often relies on supercontinuum light sources. These sources can introduce a compromised signal-to-noise ratio and increased phase noise, which may affect the precision of Doppler measurements. Some research strategies involve using NIR-OCT for reliable blood flow quantification in conjunction with vis-OCT for oximetry, thereby enabling a more thorough functional analysis of tissues like the retina [33].

DOCT detects motion by measuring the phase shift $\Delta \phi_{D}$ in the OCT signal between sequential A-lines taken at the same location with a time interval *T*. This phase shift is directly related to the axial velocity *v* of the scatterers as follows:

(5) $$v=\frac{\lambda_{0}\Delta \phi_{D}}{4\pi nT\cos\alpha },$$

where $\lambda_{0}$ is the center wavelength of the light, *n* is the tissue refractive index, and α is the angle between the beam and flow direction. Conventional DOCT is highly susceptible to phase ambiguity at shorter visible wavelengths. Shu et al. [34] addressed the pronounced phase-wrapping limitations when applied to vis-OCT by introducing a spectroscopic Doppler analysis. They exploited the linear relationship between Doppler phase shifts, $\Delta \phi_{D}\left(\tau \right)$, obtained from narrower spectral subbands (centered at wavenumber τ) and the axial flow velocity, described by the following:

(6) $$\Delta \phi_{D}\left(\tau \right)=2nTv_{\|}\tau ,$$

Velocity is then derived from slope *a* of the following relationship:

(7) $$v_{\|}=\frac{a}{2nT},$$

This slope-based calculation ingeniously bypasses the phase wrapping of individual $\Delta \phi_{D}\left(\tau \right)$ values, thereby significantly extending the measurable unambiguous velocity range in vis-OCT without resorting to conventional, often complex, phase unwrapping algorithms. A notable aspect of this spectroscopic approach is the introduction of a necessary processing step to correct for “spectroscopic phase shift discontinuity” between subbands, which is distinct from traditional phase unwrapping but essential for the accurate determination of the slope *a*.

While DOCT is a powerful technique for quantitative flow measurement, its clinical and research utility is limited by several inherent constraints [35]. Specifically, accurate velocity quantification requires precise knowledge of the Doppler angle, and the method is intrinsically insensitive to flow components that are perpendicular to the imaging beam. These limitations hinder its effectiveness in imaging complex microvascular networks with diverse vessel orientations. To overcome these challenges, OCTA was developed as a complementary, angle-insensitive approach.

#### 2.3.2. Vis-OCT Angiography (Vis-OCTA)

Vis-OCTA extends conventional OCTA into the visible spectrum, enabling dye-free, depth-resolved imaging of the microvasculature by detecting temporal changes of speckles caused by moving scatterers, primarily red blood cells [36]. Compared to conventional NIR-OCTA, vis-OCTA offers several compelling advantages. Primarily, the shorter wavelengths of visible light offer significantly higher spatial resolution compared to conventional OCTA, enabling finer delineation of microvascular structures [37]. Moreover, visible light exhibits stronger absorption by biological chromophore, particularly hemoglobin, therefore vessel contrast can be enhanced and functional imaging applications such as oximetry can be facilitated [22]. The unique scattering properties of tissues in the visible spectrum can also contribute to enhanced angiographic contrast [38].

**Figure 3:**
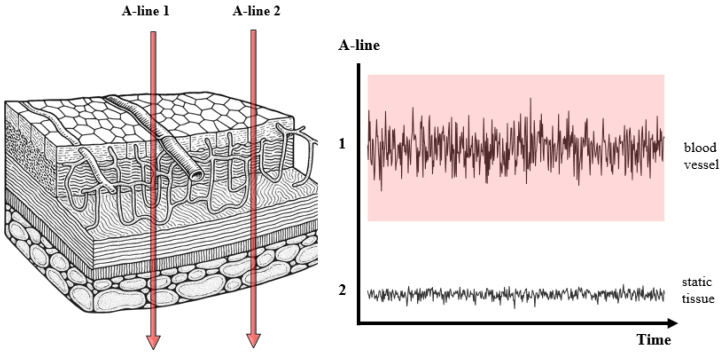
Simplified schematic of the concept of OCTA. Signals are sampled from two different locations: A-line 1 passes through a blood vessel, while A-line 2 passes through only static tissue. Dynamic changes in the OCT signal are observed from within the blood vessel over time, while the signal from the static tissue remains steady.

The fundamental mechanism of contrast generation in vis-OCTA involves repeated acquisition of OCT B-scans at the same tissue location, as shown in Figure 3. Temporal variations in the backscattered signal caused by the movement of red blood cells manifest as changes in amplitude (decorrelation) or phase [39]. In the visible spectrum, red blood cells scatter more strongly than in the NIR, which can enhance decorrelation and yield stronger motion contrast [40]. The unique speckle characteristics of visible light further influence contrast generation and may affect the performance of OCTA algorithms originally developed for NIR systems [41].

The OCT signal is naturally a complex function, consisting of amplitude and phase information, and it can be written as the equation below [42]:

(8) $$C_{OCT}\left(x,z,t\right)=I\left(x,z,t\right)e^{-i\Phi (x,z,t)},$$

where I(x,z,t) indicates the amplitude component and Φ(x,z,t) presents the phase component in an OCT signal. Typical OCTA signal processing approaches can be broadly categorized into phase-based, intensity-based, and complex signal-based methods [43]. In the context of vis-OCTA, these motion contrast-based algorithms are often adapted to leverage the higher scattering and absorption properties of visible light. Additionally, in contrast to conventional motion-based OCTA techniques, which rely on phase, amplitude, or complex signal variations, spectral contrast OCTA (SC-OCTA) was introduced by Winkelmann et al. using visible light [40]. SC-OCTA enables single-scan vessel imaging by exploiting the unique spectral signatures of blood, eliminating the need for motion contrast commonly used in traditional methods. Here, we focus on signal processing approaches specifically developed or adopted for vis-OCTA.

**Phase-based OCTA methods**: Inspired by DOCT, Phase-Variance OCT (PV-OCT) was developed to enhance sensitivity to microcirculation and reduce dependence on flow orientation. This method measures the variance of the phase difference Δϕ between B-scans repeated at the same location [44]. The flow signal is calculated as follows [43]:

(9) $$Flow_{PV}\left(x,z\right)=\frac{1}{N-1}\sum_{i=1}^{N-1}{\left[\Delta \Phi_{i}\left(x,z\right)-\frac{1}{N-1}\sum_{i=1}^{N-1}\Delta \Phi_{i}\left(x,z\right)\right]}^{2},$$

(10) $$\Delta \Phi_{i}\left(x,z,t\right)=\Phi_{i+1}\left(x,z,t+T\right)-\Phi_{i}\left(x,z,t\right)$$

where *N* is the number of B-scan repetitions, $\Phi_{i}\left(x,z,t\right)$ is the phase at location (x,z) in the *i*-th B-scan at time *t*, and *T* is the inter-B-scan time interval. This approach increases sensitivity to slower flows but sacrifices velocity information.

Due to the significant impact of phase noise on phase-signal-based OCTA—a challenge particularly exacerbated in vis-OCT systems where frequently used supercontinuum sources can introduce substantial noise—pure phase-based methods for generating OCTA images are less commonly employed in the visible light domain. Despite these challenges, efforts to enhance the robustness of phase-based vis-OCT continued. Wei et al. [45] introduced a novel bulk motion compensation method utilizing the standard deviation of phase-based OCTA and DOCT flow signals. They used the variance *v* of the OCT signal difference between two scans. By introducing a known, artificial phase shift θ into one of the signals, the variance becomes a predictable function of this θ and the unknown $\phi_{B}$:

(11) $$v\left(\theta \right)=C_{1}-C_{2}\cos\left(\Delta \phi_{B}+\theta \right)$$

where $C_{1}$ and $C_{2}$ are constants. By measuring v(θ) at four specific points, the bulk motion phase $\Delta \phi_{B}$ can be directly calculated as $\Delta \phi_{B}=atan2\left(\frac{v_{3}-v_{4}}{v_{2}-v_{1}}\right)$, where atan2 is the four-quadrant inverse tangent.

**Intensity-based OCTA methods**: Intensity-based methods measures the variance of the intensity between repeated B-scans at the same location [43].The application of intensity-based OCTA, particularly through amplitude-decorrelation algorithms such as split-spectrum amplitude-decorrelation angiography (SSADA) [10] has been pivotal in advancing vis-OCTA development and functional imaging applictions. In SSADA, decorrelation is calculated between consecutive B-scans (*N* repetitions) for each spectral subband, and the results are averaged as follows:

(12) $$\bar{D}\left(x,z\right)=1-\frac{1}{N-1}\frac{1}{M}\sum_{n=1}^{N-1}\sum_{m=1}^{M}\frac{A_{n}\left(x,z\right)A_{n+1}\left(x,z\right)}{\frac{1}{2}A_{n}{\left(x,z\right)}^{2}+\frac{1}{2}A_{n+1}{\left(x,z\right)}^{2}}$$

This averaging enhances the SNR and reduces sensitivity to bulk motion artifacts, albeit at the cost of reduced axial resolution [10,21]. Another pioneering work by Shah et al. [46] demonstrated the early utility of vis-OCTA in a preclinical setting by monitoring laser-induced choroidal neovascularization in mice. Later, Pi et al. presented a high axial resolution (1.2 µm in tissue) fiber-based vis-OCT system [47], This system was utilized for both structural and angiographic imaging in normal rat retina and chicken embryo vasculature, showcasing the ability to identify three distinct retinal capillary plexuses and the choriocapillaris in rats using SSADA after motion correction. To mitigate motion artifacts, the implemented algorithm first cropped B-scans around the center of mass to isolate the tissue region. A 2D cross-correlation was then calculated between the first cropped B-scan, f[x,z], and each subsequent *i*-th cropped B-scan, $g_{i}\left[x,z\right]$, at the same y-location. The 2D cross-correlation is defined as follows:

(13) $$\left(f*g_{i}\right)\left[\tau_{x},\tau_{z}\right]=\sum_{x}\sum_{z}f\left[x,z\right]g_{i}\left[x+\tau_{x},z+\tau_{z}\right]$$

The displacement $\left[\tau_{x},\tau_{z}\right]$ that maximized this cross-correlation value indicated the misalignment between the B-scans. Finally, the *i*-th B-scan was co-registered by shifting it according to the determined misalignment in the x (transverse) and z (axial) directions. Building on the SSADA method, vis-OCT has been applied to map retinal vasculature and oxygen metabolism [48], monitor vascular responses to intraocular pressure changes [49], and achieve single-capillary oximetry across all retinal plexuses in rats [37].

**Complex signal-based OCTA methods**: These techniques utilize both the amplitude and phase information contained within the complex OCT signal to enhance flow detection sensitivity. One of the representatives of complex signal-based OCTA techniques is optical microangiography (OMAG), which computes the difference between complex OCT signals from consecutive B-scans (*N* repetitions) after applying phase compensation methods to mitigate bulk motion artifacts [50]. The flow signal is calculated as the average magnitude of the complex differential signal as follows [51]:

(14) $$Flow_{OMAG}\left(x,z\right)=\frac{1}{R-1}\sum_{i=0}^{R-1}\left|C_{i+1}\left(x,z\right)-C_{i}\left(x,z\right)\right|$$

where $C_{i}\left(x,z\right)$ is the complex signal at location (x,z) in the *i*-th B-scan. OMAG offers high sensitivity to microvasculature and can potentially provide directional information. Yi et al. pioneered *in vivo* OMAG with vis-OCT, simultaneously achieving 3D anatomical imaging, capillary-level microvascular visualization, and $sO_{2}$ measurement [52,53]. The functional microangiography was generated by first performing motion correction on wavelength-dependent B-scans. This process enhanced the microvasculature based on speckle contrast caused by moving blood cells.

While vis-OMAG typically employs subsequent phase compensation for artifact reduction, Nam et al. [54] introduced the Complex Differential Variance (CDV) algorithm, an alternative approach designed to intrinsically suppress certain depth-correlated phase artifacts, potentially reducing errors from separate compensation steps. The CDV algorithm generates OCTA contrast by exploiting changes in both the intensity and phase of the OCT signal R(z,t). For a set of M complex A-line measurements, the CDV signal $f_{CDV}\left(z\right)$ is calculated as follows:

(15) $$f_{CDV}^{2}\left(z\right)=1-\frac{\sum_{t=1}^{M-1}\left|\sum_{k=-L}^{L}W\left(k\right)R\left(z-k,t\right)R^{*}\left(z-k,t+1\right)\right|}{\sum_{t=1}^{M-1}\sum_{k=-L}^{L}W\left(k\right)\frac{{\left|R\left(z-k,t\right)\right|}^{2}+{\left|R\left(z-k,t+1\right)\right|}^{2}}{2}}$$

where w(k) is a depth window function of length 2L+1. Addictionally, Shi et al. [55] introduced a normalized complex decorrelation algorithm that aims to achieve high-contrast angiograms by utilizing a Sigmoid-shaped curve to effectively suppress background noise and enhance vessel visibility as follows:

(16) $$g_{NDC}=\frac{1}{1+e^{10\cdot (a-0.5)}}$$

The normalized complex decorrelation value *a* is calculated from CDV methods. This $g_{NDC}$ mapping aims to better suppress static tissue background and enhance flow signals.

Wei et al. [56] proposed Phase-Stabilized Complex-Decorrelation (PSCD) angiography as an approach to enable robust, explicit pre-processing to ensure phase stability. The PSCD algorithm first applies a dedicated method to compensate for motion and stabilize phase [45]. Following phase stabilization, it calculates the OCTA signal based on the amplitude $\left(A_{n},A_{n+1}\right)$ and the stabilized phase difference $\theta_{n}^{{\,}^{\prime }}$ between two consecutive complex OCT signals $\left(C_{n},C_{n+1}\right)$ as follows:

(17) $$\theta_{n}^{\prime }\left(x,z\right)=\left|\tan^{-1}\frac{\mathrm{imag}(C_{n}\left(x,z\right)\cdot C_{n+1}{\left(x,z\right)}^{*})}{\mathrm{real}(C_{n}\left(x,z\right)\cdot C_{n+1}{\left(x,z\right)}^{*})}\right|$$

(18) $$PSCD=\sum_{n=1}^{N-1}\frac{{\left[A_{n}\left(x,z\right)+A_{n+1}\left(x,z\right)\right]}^{2}\cdot \theta_{n}^{\prime }{\left(x,z\right)}^{2}}{A_{n}^{2}\left(x,z\right)+A_{n+1}^{2}\left(x,z\right)}$$

**SC-OCTA method**: SC-OCTA is introduced as a technique enabling single-scan vessel imaging by leveraging the distinct spectral signatures of blood compared to surrounding tissue within the visible light spectrum [40]. This approach inherently obviates the need for motion-based contrast, a common requirement in traditional OCTA methods.

The underlying principle of SC-OCTA is based on the differential spectral response of hemoglobin and tissue in the 550 nm to 600 nm visible light range. Depth-resolved sample information is initially acquired by Fourier transforming the interference spectrum. Spectrally dependent OCT A-lines I(k,z) are then measured using STFT. Following calibration $I_{cal}\left(k,z\right)$, which is obtained by imaging a standard aqueous solution of latex beads to correct for wavelength-dependent variations in the system’s spectral response, the processed intensity is determined by the following:

(19) $$I\left(k,z\right)={\left(\frac{I_{samp}\left(k,z\right)}{I_{cal}\left(k,z\right)}\right)}^{2}k^{4}$$

where $I_{samp}\left(k,z\right)$ represents the STFT of the sample signal. To achieve high spectral contrast, the method employs two specific Kaiser sampling windows centered at 557 nm and 620 nm. Let I(557) and I(620) denote the 3D OCT datasets corresponding to these respective spectral windows. The SC-OCTA intensity $I_{SC-OCTA}$ is subsequently calculated by taking the ratio of the log-transformed and median-filtered intensities from these two windows as follows:

(20) $$I_{SC-OCTA}=m\left(\frac{m(\log(I(620)))}{m(\log(I(557)))}\right)$$

This ratiometric calculation effectively highlights blood vessels based on their unique spectral characteristics. A significant advantage of SC-OCTA is its reliance on these intrinsic spectral properties derived from a single OCT scan, which circumvents the motion artifacts prevalent in multi-scan, motion-dependent OCTA techniques and allows for faster image acquisition.

#### 2.3.3. Vascular Oximetry

Oxygen saturation ($sO_{2}$) is a crucial physiological parameter that reflects the metabolic status of tissues, particularly in the early diagnosis and pathological investigation of ocular diseases [33,37]. Conventional imaging techniques, such as fundus photography and photoacoustic microscopy, are limited by either imaging depth or spatial resolution, which makes it challenging to accurately quantify oxygenation within the retinal capillary network [37]. In the early 2010s, Robles et al. [57] and [58] were among the first to explore the use of vis-OCT for measurement of retinal oxygenation. In contrast to conventional methods, vis-OCT leverages the strong absorption of hemoglobin in the visible spectral range, providing superior axial resolution and enhanced spectral contrast between oxygenated and deoxygenated hemoglobin. These advantages open up new possibilities for noninvasive imaging of oxygen metabolism in microvascular structures [59].

Vis-OCT performs time–frequency analysis of A-line signals in sub-spectral windows to reconstruct depth-resolved tissue backscattering properties at different wavelengths. Based on the distinct optical absorption characteristics of hemoglobin, $sO_{2}$ is estimated using a least-squares inverse algorithm. By fitting multi-wavelength spectral data, the individual contributions of oxygenated ($HbO_{2}$) and deoxygenated hemoglobin (Hb) can be separated, thereby enabling the quantitative calculation of vascular $sO_{2}$. Specifically [59], assuming uniform concentrations of $HbO_{2}$ and Hb within a vessel, the wavelength-dependent A-line reflectance intensity I(z,λ) can be expressed by Equation (8), which is derived from the power-law model $r\left(\lambda \right)=A\lambda^{-\alpha }$ under the first-order Born approximation [60]. The effective extinction coefficients of $HbO_{2}$ and Hb are annotated by $\epsilon HbO_{2}\left(\lambda \right)$ and ϵHb(λ). By taking the natural logarithm of the normalized spectrum, a linear expression for the optical depth (OD), representing the axial position in the depth domain, can be derived using Equation (22). Then the $sO_{2}$ can be calculated as $sO_{2}=CHbO_{2}/\left(CHbO_{2}+CHb\right)$

(21) $$I\left(z,\lambda \right)=I_{0}\left(\lambda \right)R_{0}r\left(\lambda \right)e^{-2(z-z_{0})\left[c_{{\mathrm{HBO}}_{2}}\varepsilon_{{\mathrm{HBO}}_{2}}\left(\lambda \right)+c_{\mathrm{HB}}\varepsilon_{\mathrm{HB}}\left(\lambda \right)\right]},$$

where $z_{0}$ is the depth of the vascular anterior boundary, $I_{0}\left(\lambda \right)$ is the incident spectrum, $R_{0}$ is the wavelength independent reference reflectance, and $R_{\lambda }$ is the reflectance in the vessel.

(22) $$\mathrm{OD}\left(z,\lambda \right)=\ln\left(\frac{I(z,\lambda )}{I_{0}\left(\lambda \right)}\right)=-2\left(z-z_{0}\right)\left[c_{{\mathrm{HbO}}_{2}}\varepsilon_{{\mathrm{HbO}}_{2}}\left(\lambda \right)+c_{\mathrm{Hb}}\varepsilon_{\mathrm{Hb}}\left(\lambda \right)\right]-\alpha \ln\lambda +\ln\left(AR_{0}\right),$$

Spectral OD is extracted via STFT, and the fitting is formulated as a linear matrix equation as follows:

(23) $$\left[\begin{array}{c} OD(z,\lambda_{1})\\ OD(z,\lambda_{2})\\ ...\\ OD(z,\lambda_{n}) \end{array}\right]=\left[\begin{array}{cccc} \varepsilon_{HbO_{2}}\left(\lambda_{1}\right) & \varepsilon_{Hb}\left(\lambda_{1}\right) & \ln(\lambda_{1}) & 1\\ \varepsilon_{HbO_{2}}\left(\lambda_{2}\right) & \varepsilon_{Hb}\left(\lambda_{2}\right) & \ln(\lambda_{2}) & 1\\ ... & ... & ... & ...\\ \varepsilon_{HbO_{2}}\left(\lambda_{n}\right) & \varepsilon_{Hb}\left(\lambda_{n}\right) & \ln(\lambda_{n}) & 1 \end{array}\right]\left[\begin{array}{c} -2\left(z-z_{0}\right)C_{HbO_{2}}\\ -2\left(z-z_{0}\right)C_{Hb}\\ -\alpha \\ \ln(AR_{0}) \end{array}\right]$$

Achieving accurate and non-invasive measurement of blood $sO_{2}$ has consistently been the subject of research in this field. In recent years, significant progress has been made in vis-OCT-based oximetry. Pi et al. first reported a method for quantitatively measuring $sO_{2}$ in capillary segments within the three-layered vascular plexus of the rat retina—namely, the superficial vascular plexus (SVP), intermediate capillary plexus (ICP), and deep capillary plexus (DCP)—using vis-OCT. This work represents a major breakthrough in achieving capillary-level oximetry [37]. The method demonstrated excellent repeatability and sensitivity under normoxic, hypoxic, and hyperoxic conditions, indicating that capillary $sO_{2}$ may serve as a crucial physiological biomarker for assessing local retinal metabolic activity. Furthermore, the study observed a gradual decrease in capillary $sO_{2}$ along the flow path away from arterioles, suggesting that oxygen release predominantly occurs at the capillary level within the retinal tissue. Applying Wishart random-matrix theory, Qiao et al. statistically isolated the ballistic component in vis-OCT spectra via an adaptive low-rank eigenvalue selection, eliminating multiple-scattering noise without hardware modification. The method boosted retinal raster-scan SNR by 23.6 % and constrast-to-noise ratio (CNR) by 1.6-fold, greatly enhanced $sO_{2}$ accuracy, and it offered a transferable framework for other SD-OCT systems [61]. Rubinoff et al. developed adaptive spectroscopic vis-OCT (ADS-vis-OCT), a novel methodology that accurately measures retinal $sO_{2}$ by adaptively removing spectral contaminants unique to each vessel. They extracted localized spectra using STFT, then adaptively selected the signal onset depth, depth window, and system parameters for each individual vessel. By combining physical modeling with statistical regression, they effectively removed spectral contaminants and accurately estimated $sO_{2}$. Subsequently, they demonstrated the technique’s high accuracy by achieving a bias of only 1% compared to ex vivo blood gas measurements and a root-mean-squared error of 2.1% against pulse oximeter readings in human retinal arteries, along with excellent repeatability, significantly advancing vis-OCT toward clinical ophthalmic applications [62].

Overall, vis-OCT, combined with advanced image processing and spectral analysis techniques, enables noninvasive, microscale oxygenation measurement under *in vivo* conditions. This technology not only provides a new tool for understanding normal retinal metabolism but also lays the foundation for the early detection and dynamic monitoring of diseases such as glaucoma and diabetic retinopathy. Future research directions include integrating blood flow velocity measurements to achieve three-dimensional reconstruction of localized oxygen metabolic rates ($MRO_{2}$) and extending the application of this technology to clinical imaging of the human eye.

#### 2.3.4. Vis-OCT for Nanoscale Sensing

Yi et al. demonstrated that OCT can achieve sensitivity to nanoscale structural alterations in biological tissue through a technique called Inverse Spectroscopic OCT (ISOCT) [60]. Rather than directly resolving individual nanostructures, ISOCT quantifies the statistical mass-density distribution via its refractive index autocorrelation function ($C_{n}\left(\rho \right)$), which is modeled using the Whittle–Matern functional family. The core principle involves measuring the wavelength-dependent backscattering coefficient, $\mu_{b}\left(\lambda \right)$, and analyzing its spectral characteristics.

Under the Born approximation,$\mu_{b}\left(\lambda \right)$ is linked to a key structural parameter, *D* (which reflects nanoscale heterogeneity), through the following relationship:

(24) $$\mu_{b}\propto k^{4-D},$$

where *k* is the wavenumber. By extracting *D* from the measured power-law dependence of the backscattering spectrum, ISOCT can detect changes in tissue structure within its sensitivity range of approximately 30 nm to 450 nm. Therefore, ISOCT infers sub-diffractional, statistical information about tissue nano-architecture by analyzing the spectral signature of backscattered light, bypassing the conventional diffraction limits of imaging resolution. Compared with scanning electron microscopy images, ISOCT demonstrated excellent capability in quantitatively assessing structural differences between different tissue regions, such as the epithelium and stroma [22,60,63].

### 2.4. Vis-OCT Technical Challenges

#### 2.4.1. Broadband Visible Light Source

The quest for higher resolution in OCT hinges on the light source. Theoretically, an OCT system’s axial resolution improves with a shorter center wavelength and a broader spectral bandwidth, while lateral resolution is directly proportional to the center wavelength. While these principles are clear, achieving simultaneous enhancements in both axial and lateral resolution is technically demanding. It requires advanced light sources that offer both ultra-broad bandwidth and a low center wavelength, ideally within the visible spectrum.

To date, the following four main types of light sources have been employed in OCT systems, each with specific characteristics and applications: superluminescent diodes (SLDs) [64,65], ultrafast lasers [21], swept-source lasers [16], and supercontinuum (SC) sources. SLDs are prevalent in commercial OCT systems due to their cost-effectiveness and reliability. However, their typical bandwidths and NIR spectral range (e.g., 670–1600 nm in some contexts, though visible SLDs exist with narrower bandwidths) limit their use in ultrahigh-resolution vis-OCT [66]. Nevertheless, vis-OCT implementations using SLDs remain feasible when combined with complementary strategies such as deep learning-based reconstruction or spectral estimation techniques to enhance axial resolution [64,65,67]. Ultrafast lasers (e.g., Ti:Sapphire lasers [21]) can access visible wavelengths through nonlinear effects but face challenges in spectral efficiency and effective bandwidth. While highly effective for high speed deep tissue imaging in NIR-OCT, developing practical frequency-swept lasers in the visible range remains a key challenge. However, indirect conversion methods are emerging [16], with ongoing efforts focused on improving output power and sensitivity for vis-OCT applications.

Compared to other light sources, SC sources based on nonlinear spectral broadening in photonic crystal fibers (PCFs) demonstrate unique advantages as follows: they provide ultra-broad spectral coverage (400-2500 nm) while maintaining exceptional spatial coherence. This breakthrough enables vis-OCT systems to achieve ultrahigh axial resolution, establishing SC sources as the most effective solution so far for vis-OCT.

#### 2.4.2. Dispersion Management

Dispersion refers to the phenomenon where different wavelengths of light propagate at distinct velocities, causing phase delays during transmission [68]. Vis-OCT typically utilizes SC light sources to obtain higher resolution and richer spectral information. However, the implementation of such broadband light sources can introduce significant dispersion, leading to broadening of point spread function (PSF) and degradation of axial resolution [69]. Vis-OCT is more sensitive to dispersion compared to NIR-OCT. This increased susceptibility arises from the following two key factors: Firstly, optical glass exhibits stronger refractive index variations within the visible spectrum [70]. Secondly, the elevated scattering and absorption coefficients in the visible relative to NIR range result in differential propagation paths and effective penetration depths of different wavelengths through biological tissues [71], thereby amplifying the impact of dispersion on image quality.

Dispersion management in vis-OCT generally adopts a dual compensation framework integrating hardware enhancements with algorithmic corrections. Hardware compensation aims to directly balance or minimize dispersion within the optical path through strategic optical design and the incorporation of physical components. In terms of optical design, fundamental methods for balancing dispersion include the use of low-dispersion achromatic lenses and the precise matching of optical fiber lengths in the reference and sample arms. Some studies also describe the use of free-space configurations to avoid additional dispersion introduced by fiber couplers [72,73]. Further hardware-based dispersion compensation primarily involves either introducing dispersive media into the reference arm or employing specialized devices such as prism pairs [74], tilt gratings [75], phase-scanned delay lines [76], acousto-optic modulators [77], and dual-fiber stretchers [78]. These approaches may exhibit limitations including substantial implementation costs, increased system complexity, and constrained compensation bandwidth. Algorithmic approaches provide enhanced flexibility and adaptability beyond hardware compensation. A widely used technique involves applying phase correction functions (typically polynomial-based) to spectral interferograms for neutralizing group velocity dispersion and higher-order effects [68]. These corrections are optimized through automated iterative algorithms that adjust phase terms to maximize image sharpness metrics such as axial PSF peak intensity, enabling adaptive compensation for system and sample variations. Additionally, novel approaches such as subband and sub-image correlation analyses were proposed to decompose OCT data into different spectral and spatial components to address depth and position dependent dispersion of vis-OCT [79]. Another important strategy involves measurements of single arbitrary mirror reflections, which extract dispersion-induced phase delays through Hilbert transform and spectral analysis, offering simplified operation and robust compensation for high-order dispersion across different OCT systems [80].

#### 2.4.3. Limited Depth Penetration

In biological tissues, visible light exhibits strong attenuation, limiting the effective imaging depth of visible-light OCT [17]. Beyond biological tissue attenuation, the imaging depth of SD-OCT is constrained by the spatial resolution of the spectrometer, with the maximum imaging depth determined by the Nyquist criterion [17]:

(25) $$z_{max}=\frac{\lambda_{0}^{2}}{4\cdot \delta_{S}\lambda },$$

where $\delta_{S}\lambda$ is the wavelength interval between adjacent detector pixels. Using a detector with a smaller wavelength interval can help compensate for the sensitivity roll-off in visible-light imaging. In practice, multiple factors limit the actual depth penetration of vis-OCT, including strong scattering and absorption at short wavelengths, lower permissible exposure levels, and increased speckle noise. One effective approach is to implement linear-in-wavenumber sampling to mitigate the sensitivity roll-off by incorporating a prism combination into the optical path of conventional spectrometers [81]. This redistributes the spectrum to uniform wavenumber intervals at the detection array of the spectrometer, ensuring uniform Fourier-domain sampling. Additionally, in vis-OCT systems, the Fourier transform of the real-valued spectral signal generates complex conjugate symmetry, introducing mirror artifacts around the zero delay [82], further compromising deep tissue imaging. Methods including Hilbert phase microscopy [83], phase-shifting interferometry [84], and off-axis holography [85] have been proposed to suppress the complex conjugate artifact and double the image depth. Alternatively, acquiring both in-phase and quadrature components of the Fourier-domain signal resolves depth degeneracy [86,87,88,89,90].

#### 2.4.4. System Noise Reduction

The relatively low SNR in vis-OCT arises from the stronger scattering and absorption of visible light in tissue, reduced optical power due to safety limits, and increased sensitivity to noise from SC sources. Since the scattering properties of biological tissues and the safety exposure thresholds are difficult to alter, most efforts to enhance SNR in vis-OCT have focused on improving detection sensitivity by suppressing system noise. There are three main types of noise contributing to the total source of an OCT system, outlined as follows: detector noise (including read noise and dark noise), shot noise, and excess noise from the light source [91]:

(26) $$\sigma_{noise}^{2}=\sigma_{r+d}^{2}+\sigma_{shot}^{2}+\sigma_{E}^{2},$$

An ideal OCT system operates in the shot-noise limit, where the dominant noise source originates from the quantum uncertainty of photons rather than system-induced noise [92]. Excess noise is one of the primary factors affecting imaging quality and system sensitivity in SC source-based vis-OCT systems, with its main source identified as the relative intensity noise (RIN) from the light source [93,94]. RIN manifests as random fluctuations in spectral intensity between pulses, caused by temporal instabilities during the SC generation process [95,96]. A straightforward approach to suppress RIN is to increase the spectrometer camera’s exposure time, thereby averaging out fluctuations in output intensity over time [38]. However, this method is highly sensitive to motion and increases the optical exposure to the sample, which is undesirable for many *in vivo* imaging applications. Alternatively, SC generation in all-normal dispersion (ANDi) fibers has shown promising shot-noise-limited behavior in the far-red spectral region [97]. This low-noise characteristic is primarily attributed to the spectral broadening mechanisms within ANDi fibers, which are dominated by self-phase modulation (SPM) and optical wave breaking. These processes, unlike the soliton-related dynamics prevalent in anomalous dispersion fibers, tend to preserve coherence and avoid the significant amplification of input noise, leading to a more stable and quieter SC generation. Yet, further advancements are needed to extend the applications to shorter wavelength vis-OCT.

Balanced detection, which suppresses common-mode noise by differentially processing complementary signals from dual detectors, has been extensively utilized in SS-OCT systems to enhance SNR [15]. Researchers further extended this technique to vis-OCT by employing dual spectrometers to simultaneously capture two spectra, thereby reducing the RIN floor [27,38]. For example, Rubinoff et al. [38] implemented sub-pixel level calibration between two spectrometers by exploiting the correlation in RIN between the two channels. With the calibrated dual-spectrometer setup, the balanced detection demonstrated significantly improved noise suppression, achieving up to 20.5 dB reduction in the vis-OCT system’s noise floor. To reduce system cost, some studies have explored single-spectrometer noise suppression schemes. Wang et al. [98] achieved SNR enhancement by $\sqrt{M}$-fold RIN reduction through spectral bandwidth broadening via multi-channel input, achieving high sensitivity with low cost. Wan et al. [99] implemented balanced detection using angularly multiplexed spectra on a single spectrometer, suppressing DC and AC noise and improving SNR by up to 6 dB.

These advancements underscore the critical role of RIN suppression in realizing high-performance vis-OCT imaging. While dual-spectrometer balanced detection remains the most effective strategy to date, emerging single-spectrometer approaches offer promising trade-offs between performance, cost, and complexity. Continued innovation in both light source engineering and detection schemes will be essential for further improving SNR and enabling broader preclinical and clinical applications of vis-OCT.

## 3. Biomedical Applications of Vis-OCT

### 3.1. Applications in Ophthalmologic Imaging

Ophthalmic imaging remains the most mature and clinically successful application for OCT. The eye’s inherent transparency provides an ideal window for optical interrogation, making OCT an indispensable tool for visualizing and quantifying ocular structures. Numerous ocular pathologies, including diabetic retinopathy [100], glaucoma [101], and macular degeneration [102], manifest early-stage alterations in retinal architecture and hemodynamic characteristics. Vis-OCT employs shorter wavelengths compared to conventional NIR-OCT, and inherently offers advantages in axial resolution and provides distinct scattering and absorption contrasts, which is crucial for applications such as oximetry. These technical superiorities position vis-OCT as a powerful imaging modality for both fundamental ophthalmic research and clinical diagnostics, offering comprehensive qualitative and quantitative assessments critical for early disease diagnosis and management. A significant body of vis-OCT research in ophthalmology has utilized animal models, which, due to their anatomical and genetic similarities to humans, can effectively simulate human eye diseases, thereby advancing our understanding of disease mechanisms and the development of treatment methods.

#### 3.1.1. High-Resolution Structural Imaging

**Figure 4:**
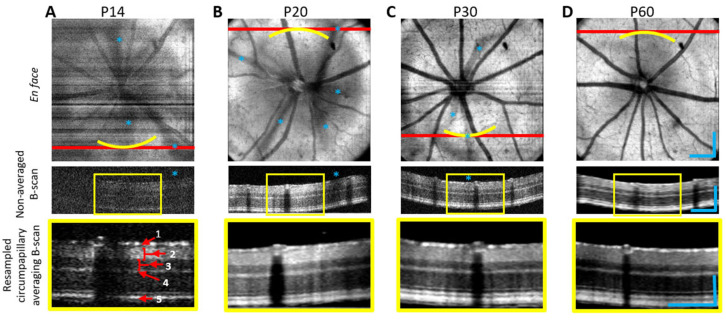
Representative vis-OCT images illustrating postnatal retinal development in mice. (**A**) P14 (right after eye-opening); (**B**) P20 (one week post-eye-opening); (**C**) P30 (two weeks post-eye-opening); (**D**) P60 (mature adults). Top: *en face* projections with red lines indicating the location of corresponding non-averaged B-scans (middle). Yellow curves and boxes denote the circumpapillary paths used for resampled B-scan averaging (bottom). Visible light significantly improves layer contrast, especially in early-stage eyes with prominent hyaloid vessel shadows (blue asterisks). Scale bar: 200 μm. Reprinted from [103], with permission from Elsevier.

As previously noted, vis-OCT employs shorter wavelength light with superior resolution compared to NIR-OCT systems which enables ultrahigh resolution retinal imaging. Multiple research groups have conducted research on rodent models to validate the enhanced resolution and improved contrast of vis-OCT to facilitate superior visualization of the intricate microarchitecture of the retina [25,63,103,104]. For instance, Chauhan et al. [104] utilized vis-OCT to visualize sublaminar structures within the ONL and OPL of the mouse retina, identifying periodic reflectivity striations and synaptic subbands that exhibited age-dependent alterations, thereby revealing microstructural hallmarks of retinal degeneration. Similarly, Beckmann et al. [103] applied vis-OCT to longitudinally image the developing mouse retina from eye-opening to maturity, and introduced a resampled circumpapillary B-scan averaging technique to overcome optical shadowing from hyaloid vessels, enabling reliable delineation of retinal layers even at early developmental stages (Figure 4). These studies exemplify the capacity of vis-OCT not only to enhance anatomical detail beyond the reach of conventional NIR-OCT, but also to support quantitative assessments of retinal development and pathology across time.

Dual-channel imaging integrating vis-OCT and NIR-OCT is also a common strategy used to combine the high-resolution and spectral contrast of visible light with the deeper penetration and higher sensitivity of NIR imaging, enabling complementary structural and functional imaging through different retinal layers [21,63]. Chen et al. [63] conducted a comparative study using both vis-OCT and NIR-OCT for mouse retinal imaging, and they have found that vis-OCT has limited capability to image structures beneath the RPE due to its low penetration depth. Vis-OCT has also been used in preclinical studies to retrieve the distribution of rhodopsin [105], which can help assess functional rod photoreceptors.

**Figure 5:**
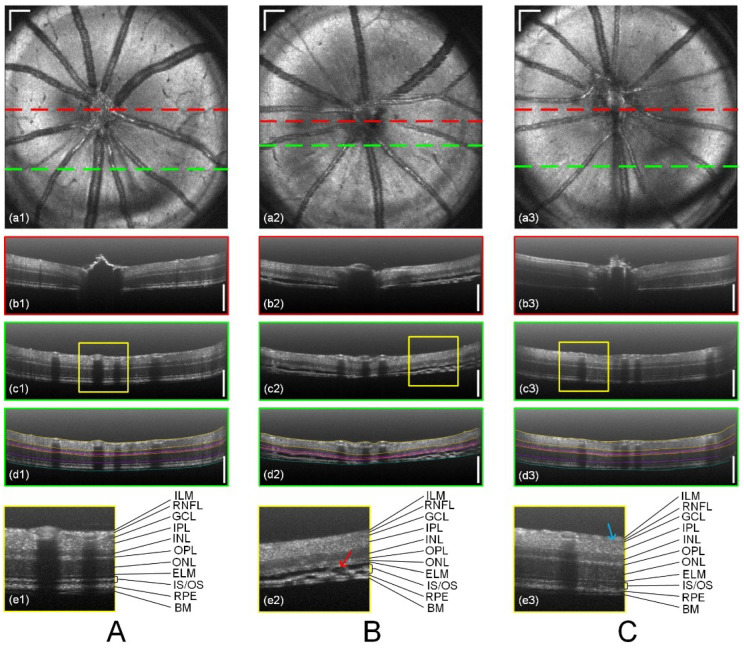
*En face* and B-scan images of mouse retina. Representative OCT images of (**A**) control, (**B**) RP, and (**C**) glaucoma mouse retina. Scale bar: 200 μm. Abbreviations: RP: retinitis pigmentosa; ELM, external limiting membrane; (**a1**–**a3**): Red (optic nerve head, (**b1**–**b3**)) and green (blood vessel cross sections, **c1**–**c3**) dashed lines indicate two distinct B-scan frames, corresponding to the colored borders of their corresponding B-scans below. (**d1**–**d3**): Retinal layer segmentation. (**e1**–**e3**): Magnified views of yellow solid-line regions in (**c1**–**c3**). (**e2**): Red arrow: lesions. (**e3**): Blue arrow: GCL. Reproduced with premission from [106], © Optical Society of America.

High-resolution structural imaging using vis-OCT has been extensively applied to various rodent models of retinal diseases. Gu et al. [106] developed a vis-OCT system integrating high-resolution imaging, GPU-accelerated computation, and automatic retinal layer segmentation, enabling 3D high-definition imaging of the mouse retina, along with blood flow and $sO_{2}$ measurements. They observed retinal thinning and damage to the ONL and IS/OS in retinal degeneration models and reduced signal in the ganglion cell layer in glaucoma models (Figure 5).

Additionally, vis-OCT can also be used in anterior segment imaging. Zhang et al. [107] applied vis-OCT to the anterior segment and introduced a compound circumlimbal scanning strategy that enabled comprehensive visualization of Schlemm’s canal and the adjacent limbal vasculature in mice. By analyzing morphology from both longitudinal and cross-sectional perspectives, they demonstrated a negative correlation between SC dimensions and intraocular pressure. Later, the same group [108] applied a robotic vis-OCT system to maintain stable, perpendicular illumination of Schlemm’s canal, achieving high-resolution circumferential imaging of aqueous humor outflow pathways. They quantified SC responses to pilocarpine treatment and found significant, segmental volume increases compared to controls.

**Figure 6:**
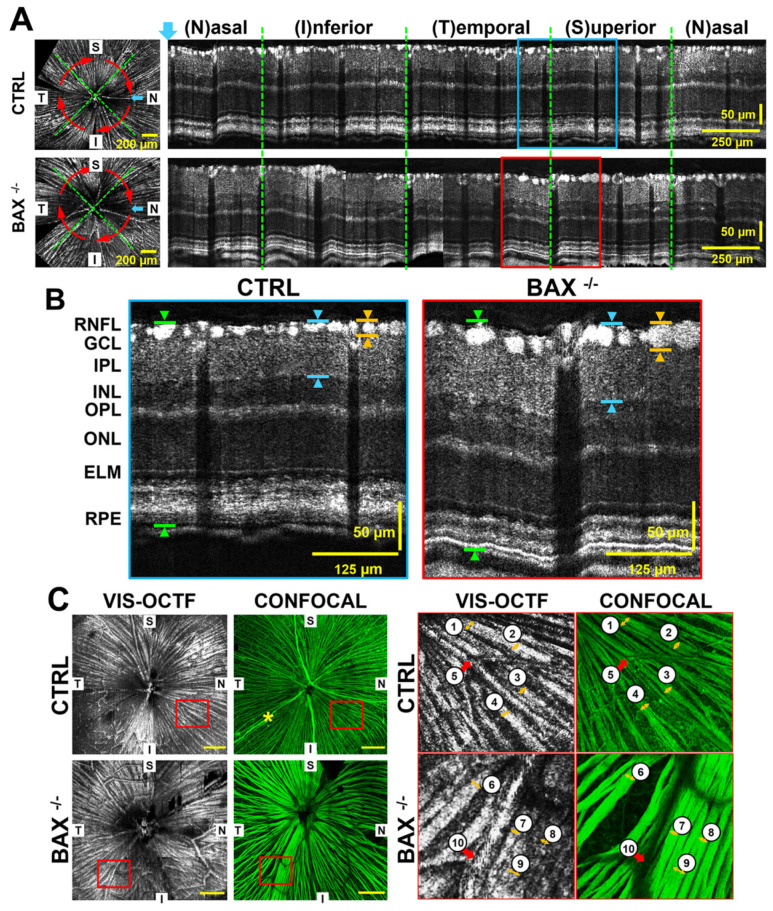
Vis-OCT fibergraphy (Vis-OCTF) reveals structural differences in the RGC axon bundles of BAX${\,}^{-/-}$ mice. (**A**) Example fibergrams (left panels) and circumpapillary B-scans (right panels) of littermate controls (CTRL) and BAX ${\,}^{-/-}$. The blue arrow highlights the left-most A-line of circumpapillary B-scans, and the red arrows indicate the direction and location of reconstructed circumpapillary B-scans. (**B**) Magnified view of the boxed regions indicated in panel A. The green arrows exemplify retinal thickness measurements, the blue arrows exemplify the GCIPL measurements, and the orange arrows exemplify RGC axon bundle measurements. (**C**) Comparing mouse RGC axon bundle organization between CTRL and BAX${\,}^{-/-}$ retinas using *in vivo* vis-OCTF and ex vivo confocal microscopy imaging of flat-mounted retinas immunostained for RGC axons (left panel) and magnified view of highlighted areas (red squares) in left panel (right panel). Numbers 1–10 denote different fiber bundles or blood vessels. Red arrows indicate blood vessels, while yellow arrows indicate nerve fiber bundles. The yellow asterisk marks a representative blood vessel. Reprinted from [109], licensed under CC BY 4.0.

Vis-OCT can also be used to quantify the retinal gangalion cell axon bundles. In 2020, Miller et al. reported a vis-OCT fiber imaging technique (vis-OCTF) [26]. Vis-OCTF is generated following the steps of RNFL segmentation, computation of the difference-from-mean metric, thresholding, and average intensity projection. Subsequently, Grannonico et al. [109] validated vis-OCTF’s ability to quantify RGC axon bundle changes in different mouse models. As shown in Figure 6, their work demonstrated that vis-OCTF could clearly identify the resulting structural differences, revealing that BAX${\,}^{-/-}$ mice have a significantly thicker RGC axon bundle layer and wider, more densely organized bundles compared to control animals. In the same study, the authors also used vis-OCTF to track RGC damage following ONC injury. In 2024, the width, height, and cross-sectional area of individual RGC bundles were quantified for the first time, and vis-OCTA was applied to observe the retinal microvasculature of tree shrews [110].

#### 3.1.2. Retinal Oximetry

The metabolism of retinal neurons relies on immediate oxygen supply [111]. Abnormal oxygen supply can lead to retinal dysfunction and cell degeneration [112], which is commonly observed in diseases such as diabetic retinopathy, glaucoma, and age-related macular degeneration. Early studies have shown that changes in hemodynamic varations and $sO_{2}$ often precede structural alterations in ocular diseases [113,114], and such oxygen metabolism can further impair retinal function through hypoxia-sensitive pathways. Therefore, accurately assessing the $sO_{2}$ and retinal metabolic rate of oxygen ($rMRO_{2}$) via vis-OCT may provide a sensitive biomarker for the early diagnosis and monitoring of retinal disease progression.

Following the initial theoretical proposals and *in vivo* demonstrations of retinal oximetry using visible light [115,116,117], Yi et al. successfully implemented the first vis-OCT system capable of quantifying retinal $sO_{2}$ *in vivo* [22]. Later, the same group [118] developed a vis-OCT system for simultaneously measuring retinal blood flow and $sO_{2}$ to quantify $rMRO_{2}$ *in vivo*. In a graded systemic hypoxia experiment using rats, they observed significant vasodilation, increased blood flow, and elevated $rMRO_{2}$, indicating that the retinal circulation actively compensates for reduced choroidal oxygen supply. In a graded systemic hypoxia experiment in rats, they observed significant vasodilation, increased blood flow, and elevated $rMRO_{2}$, indicating that the retinal circulation actively compensates for reduced choroidal oxygen supply. In 2018 [33], they further developed a dual-band dual-scan inverse spectroscopic OCT (D2-ISOCT) system, integrating visible and NIR channels to simultaneously measure capillary-level $sO_{2}$, arteriolar flow, oxygen delivery rate ($drO_{2}$), $MRO_{2}$, and nanoscale tissue structure (ΔD) *in vivo*. The system applies STFT to visible spectra, using Beer-Lambert and Mie theory to estimate $sO_{2}$, and employs spatial averaging and temporal accumulation to enable single-capillary oximetry. D2-ISOCT is also sensitive to ultrastructural changes at the scale of 30–450 nm, such as collagen crosslinking observed during wound healing, indicated by a significant increase in ΔD. This approach overcomes key limitations of conventional OCT and establishes a multimodal platform for assessing microvascular function and tissue remodeling, marking a shift toward integrated structural-functional imaging at nanoscale level.

Such blood oxygen analysis capacity has also been extended toward disease diagnosis and therapeutic monitoring, enabling earlier detection of retinal pathophysiology and assessment of microvascular dysfunction before structural damage becomes evident [30,31,32,48,53,59,106,119]. Soetikno et al. [31] analyzed the 50/10 oxygen-induced retinopathy rat model to simulate the retinopathy of prematurity. The results revealed a 61% decrease in inner retinal oxygen delivery and a 59% reduction in oxygen metabolism, demonstrating that vascular loss and neuronal damage jointly disrupt retinal oxygen balance. Liu et al. [30] employed type-1 diabetic Akita/+; TSP1−/− mice as a model of early-stage diabetic retinopathy to longitudinally quantify retinal $sO_{2}$, blood flow, and calculate the retinal oxygen metabolic rate ($IRMRO_{2}$). While arterial $sO_{2}$, blood flow rate, and vessel diameter remained stable, venous $sO_{2}$ progressively declined with age, resulting in a significant 41.3% increase in $rMRO_{2}$. Histological analysis showed no retinal structural changes, suggesting that metabolic dysfunction precedes microvascular damage, and elevated $IRMRO_{2}$ may serve as an early functional biomarker of DR. Pi et al. [119] conducted longitudinal imaging to assess retinal changes following optic nerve transection (ONT) in rats. At 4 weeks post-ONT, both arterial and venous $sO_{2}$, along with $sO_{2}$ in superficial and deep capillary plexuses were significantly reduced, despite unchanged vessel density. These findings suggest that vascular oxygenation impairment may precede or occur independently of anatomical vascular loss. In another study, Gu et al. [106] reported increased venous $sO_{2}$, reduced arteriovenous $sO_{2}$ difference, and decreased retinal blood flow in both MNU-induced retinal degeneration and NMDA-induced glaucoma mouse models, indicating hemodynamic and metabolic disruptions in both conditions.

In summary, vis-OCT oximetry shows great potential to be a early biomarker in various ophthalmic disease models. Different diseases present specific changes in oxygenation patterns: for example, the DR Model shows an increase in metabolic rate in the early stage [30], while glaucoma manifests a decrease in oxygen consumption [106]. Integrating oxygen metrics with structural parameters such as retinal thickness and vessel density enables a more comprehensive and robust assessment of disease status and progression.

#### 3.1.3. Clinical Application in Ophthalmology

Following the successful demonstration of structural and functional imaging using vis-OCT in rodent models, several research groups began translating the technology into clinical applications.

One of the main motivations to the clinical application of vis-OCT lies in the ability to visualize various sublayers of human retina with unprecedented high-resolution [23,27,29,64,120,121]. After the first demonstration of human retinal imaging by Yi et al. [23], several research groups have initiated their own efforts to explore and expand the clinical potential of vis-OCT. Chong et al. [29] developed a vis-OCT system that clearly resolved fine structural details of the retinal layers, including the NFL, GCL, IPL, INL, and OPL. Recent work by Zhang et al. [120] advanced this direction further by resolving subcellular reflectivity patterns within band 4, consistently observing two foveal and three extra hyper reflective foveal zones in all human eyes. They also introduced a correction method for modeling multiple scattering originated from the RPE to improve the Gaussian fit for the BM intensity profile and reduce the coefficient of variation of BM thickness measurement *in vivo*. Their findings support the notion that the hyperreflective structures within band 4 arise predominantly from intracellular melanosomes in the RPE, offering new insights into light-scattering mechanisms at subcellular resolution.

Beyond structural imaging, vis-OCT has been successfully adapted for retinal oximetry in human subjects [24,62,122,123,124]. Accurately extracting the true blood spectrum without noise and artifacts, especially for *in vivo* human eye imaging, is the core challenge faced by vis-OCT blood oxygen measurement. Researchers have proposed different methods to improve the accuracy of vis-OCT oximetry in human eyes. Song et al. [122] introduced vis-OCTA for localized retinal microvascular oximetry in humans. By narrowing the system’s spectral bandwidth and optimizing scanning parameters, they achieved high-speed imaging and stable $sO_{2}$ measurements within a compact field of view. The method showed high intra and intersession repeatability and visualized microvascular oximetry with fine laminar detail, particularly in metabolically active zones. Another study by Rubinoff et al. [62] further developed an adaptive spectroscopic vis-OCT to improve the accuracy of retinal $sO_{2}$ measurements. They identified spectral contaminants from both the imaging system and ocular media as a major source of error, and introduced a 12-step iterative process to remove both system- and sample-dependent spectral contaminants.

Vis-OCT has been further applied to evaluate the ocular pathological changes of living patients, such as glaucoma, DR, retinal vein occlusion and assessment of drug toxicity [123,125,126,127].

**Figure 7:**
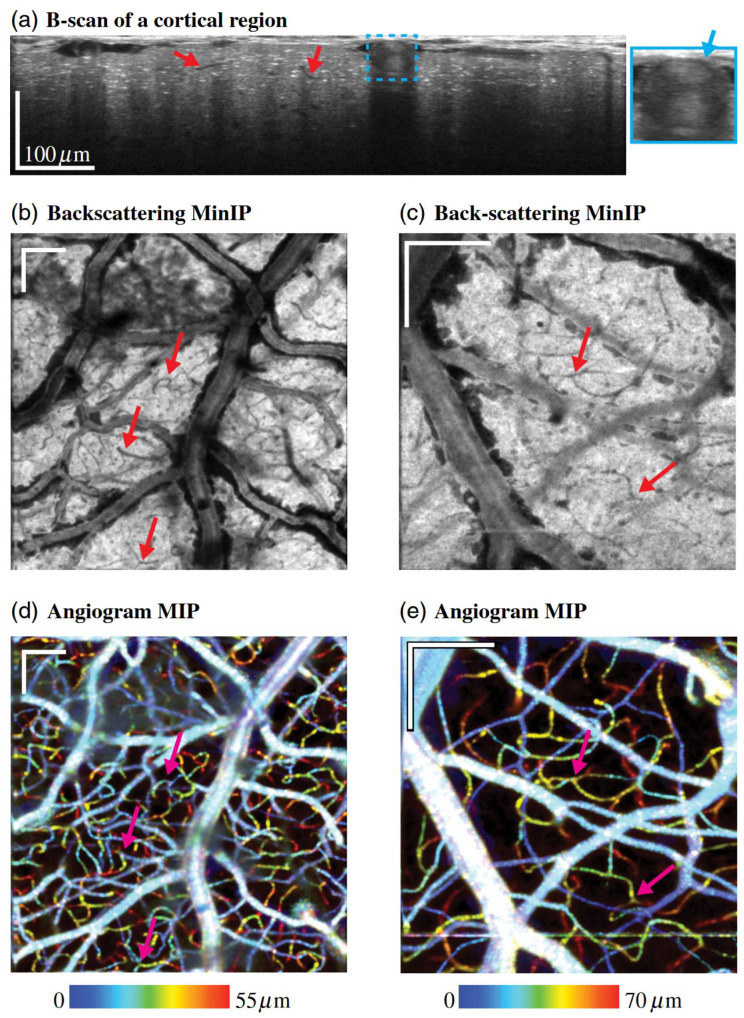
Extended-focus *in vivo* vascular imaging with visible light optical coherence microscopy (xf-visOCM): owing to the high resolution of the xf-visOCM system, the microvasculature can be resolved both in the backscattering tomograms and in the angiograms. (**a**) A B-scan covering the first ~100 μm in depth of the cortex reveals large vessels at the surface and capillaries that can be resolved as dark elongated structures, as pointed by the red arrows. Moreover, a close-up on the large caliber vessel reveals a thin dark cell-free layer below the vessel membrane (blue arrow). Vascular structures can be visualized by either performing a minimum intensity projection (MIP) on the static backscattering, in (**b**,**c**), or an MIP on the angiogram, (**d**,**e**). Similar features are highlighted between the two visualizations by red arrows in (**b**,**c**) and pink arrows in (**d**,**e**). Scale bars: 100 μm. Reproduced from [133], licensed under CC BY 4.0.

**Glaucoma**: Vis-OCT offers new perspectives for early glaucoma diagnosis and monitoring. Ghassabi et al. [125] used speckle-reduced vis-OCT to quantify IPL sublayers in healthy and glaucomatous eyes. They found significant thinning of the entire IPL in glaucoma, primarily in the middle sublayer L2, which may reflect early dendritic degeneration of retinal ganglion cells. Furthermore, Song et al. [122] demonstrated that vis-OCT derived peripapillary RNFL (pRNFL) reflectivity and a novel parameter, peripapillary vessel number (pVN), were significantly better at distinguishing glaucoma suspect/preperimetric glaucoma (GS/PPG) eyes from normal eyes than standard thickness measurements of the RNFL or macular ganglion cell-inner plexiform layer (GCIPL) by NIR-OCT. This suggests vis-OCT can detect nanoscale changes in the RGC axon cytoskeleton before bulk axon loss and measurable thinning occur. Retinal oximetry via vis-OCT has also shown altered macular venous $sO_{2}$ and reduced oxygen extraction in glaucoma patients, indicating its potential as an early diagnostic metric. A dual-channel vis-OCT system was used to quantify macular $sO_{2}$ in normal, suspect/pre-perimetric glaucoma, and perimetric glaucoma subjects. Their results revealed a progressive decline in macular arterial $sO_{2}$, arteriovenous $sO_{2}$ difference, and oxygen extraction, along with an increase in venous $sO_{2}$ as glaucoma severity advanced. Importantly, venous $sO_{2}$, and arteriovenous $sO_{2}$ difference showed significant differences between normal and suspect/pre-perimetric eyes, suggesting their potential as early biomarkers for glaucoma before structural or functional damage becomes evident (Figure 7).

**Diabetic retinopathy**: As mentioned, vis-OCT’s oximetry capabilities are highly relevant for DR, allowing quantification of retinal hypoxia, oxygen delivery, and metabolic rates, which are key factors in DR pathophysiology [30]. Wang et al. [123] used a vis-OCT- and NIR-OCT-integrated system to image healthy eyes and eyes with diabetic retinopathy, along with central retinal vein occlusion and sickle cell retinopathy eyes, revealing distinct vascular abnormalities and altered $sO_{2}$ levels.

**Assessment of drug toxitcity**: Another study conducted by Garg et al. [127] used vis-OCT to assess photoreceptor outer segment reflectivity in patients taking hydroxychloroquine. They found that attenuation of subband 3i was a consistent and early marker of retinal toxicity, even when conventional OCT appeared normal.

Additionally, cross-specie comparisons of retinal sturctures using vis-OCT have also gained attention to better understand specie-specific retinal architecture and its relevance to disease modeling. A study by Grannonico et al. [128] employed high-resolution vis-OCT to compare retinal structures in humans, tree shrews, and mice. The results demonstrated that tree shrews share key retinal features with human, such as a dense RNFL, thick GCL, and multilayered IPL. Unlike the thinner, rod-dominated mouse retina, tree shrew may be a more anatomically relevant model for optic nephropathy research.

### 3.2. Emerging Imaging Domains of vis-OCT

In addition to ophthalmic applications, vis-OCT has demonstrated its potential value in various imaging domains, with the research mainly conducted using rodent models.

**Figure 8:**
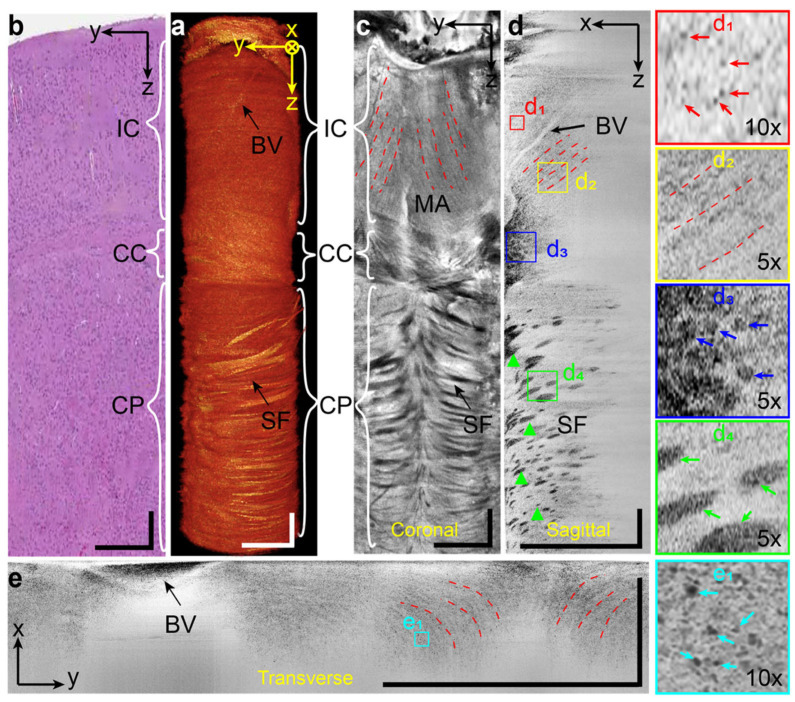
*In vivo* vis-OCT endoscopic imaging of a mouse brain with a depth of 7.2 mm. (**a**) Reconstructed 3D image of the mouse brain, showcasing distinguishable brain regions such as isocortex (IC), corpus callosum (CC), and caudate putamen (CP). One major blood vessel (BV) and striatopallidal fibers (SF) can be visualized in the isocortex and caudate putamen, respectively. (**b**) The corresponding hematoxylin and eosin (H&E) histology image. (**c**) Coronal (y-z) *en face* projection image, highlighting the myelinated axon (MA) fibers (red dashed lines) and SF (black arrow). (**d**) Sagittal (x-z) *en face* projection image with MA fibers (red dashed lines), a major blood vessel (black arrow), and the SF in the caudate putamen (green triangles). Insets d1-d4 display enlarged boxed regions: neuronal cell bodies (red arrows, **d1**), MA fibers (red dashed lines, **d2**), nerve fiber bundles (blue arrows, **d3**), and SF (green arrows, **d4**). (**e**) Transverse (x-y) *en face* projection image, featuring MA fibers (red dashed lines) and a blood vessel (black arrow, strong OCT signal attenuation). Inset (**e1**) provides an enlarged view with neuronal cell bodies indicated by the cyan arrows. In the coronal (y-z) plane, the *en face* projection begins 60 μm from the endomicroscope’s glass capillary and extends 60 μm deep. In the sagittal (x-z) plane, it starts at a depth of 1696 μm and projects 220 μm deep. In the transverse (x-y) plane, it initiates at 2000 μm from the brain surface and projects 200 μm deep. The projection was generated using mean-intensity projection. The scale bars represent 500 μm and apply to all the images. Reproduced from [140], licensed under CC BY 4.0.

**Brain tissue imaging**: Although the brain tissue does not possess the anatomical conditions for direct external imaging like the eye, its complex microcirculation system, dynamic oxygen metabolism, and pathological neurodegenerative progression share similar monitoring needs with the retina. With the aid of special procedures such as craniotomy, vis-OCT demonstrates significant potential in the study of brain microcirculation and oxygen metabolism, offering a novel visualization tool for investigating the pathological mechanisms of stroke [32,72,129,130,131,132,133]. To improve functional evaluation accuracy, Chen et al. [130] significantly improved the accuracy of $sO_{2}$ measurements through a dual-depth sampling strategy, revealing the diameter-dependent vasodilation in the penumbra following ischemia and the imbalance in oxygen metabolism. In a foundational study, Chong et al. [32] integrated Doppler blood flow and hemoglobin spectral analysis to simultaneously measure cerebral blood flow, oxygen extraction fraction, and oxygen metabolism rate for the first time, elucidating the dynamic equilibrium between cerebral blood flow and oxygen metabolism. The same group later developed a dynamic scattering analysis method, achieving simultaneous quantitative measurement of $sO_{2}$ and total hemoglobin concentration, and validated the technology’s high sensitivity to metabolic dynamics through cardiac arrest experiments [129].

vis-OCT-based high resolution microscopy has also been reported in brain tissue imaging, achieving fine structure imaging including fibers and cell bodies at sub-micron resolution [133,134]. For example, Marchand et al. [133] leveraged visible-to-NIR wideband illumination and extended-focus optical coherence microscopy to resolve fine cortical features *in vivo*, including capillaries, myelinated axons, and neuronal somas. As shown in Figure 8, both large pial vessels and individual capillaries can be clearly identified in the backscattering tomograms, while angiographic reconstructions further enhance vascular contrast and continuity. Notably, the system reveals fine structural details such as the dark, elongated profiles of capillaries and the presence of a thin, cell-free layer adjacent to vessel walls, highlighting its ability to capture subtle morphological features across multiple cortical depths. Furthermore, the spectroscopic capabilities of vis-OCT have been shown to enable the direct visualization of amyloid plaques in the brain, providing a promising endogenous biomarker for the early detection and diagnosis of Alzheimer’s disease [73,131,135].

**Skin imaging**: Revin et al. [136] demonstrated the application of vis-OCT for non-invasive imaging of the stratum corneum in non-palmar human skin. B-scan images revealed the stratum corneum as a distinct hypoechogenic dark layer beneath the skin surface, consistent with prior findings in palmar skin using conventional OCT. Moreover, the optical appearance of the stratum corneum was found to be strongly dependent on its hydration state, becoming brighter after occlusive hydration. This highlights the potential of vis-OCT for assessing epidermal barrier function and water content *in vivo*.

**Endoscopic imaging**: Endoscopic OCT has long focused on the NIR spectral range, achieving a series of breakthroughs in *in vivo* microscopic imaging of luminal organs such as the cardiovascular, gastrointestinal, and respiratory tracts. However, the inherent limit of the achievable resolution with relatively long wavelengths of NIR light has driven the exploration of OCT operating in the visible-light range. Early studies primarily focusing on large-diameter (>5mm) probes for superficial luminal tissue imaging [137,138]. Duan et al. [137] proposed and validated a 15 mm-diameter endoscopic vis-OCT system, achieving high-resolution *in vivo* imaging of microlesions and tissue layers in a rhesus macaque vaginal model, providing a new approach for noninvasive screening of female reproductive tract diseases and mucosal barrier assessment. Later, a 6 mm pullback-rotational probe was developed by Winkelmann et al. [138], combining high axial resolution (1.1 μm) in the visible range (500–695 nm) with inverse spectroscopic OCT for nanoscale structural analysis. Validated through *in vivo* human skin imaging and microsphere scattering experiments, the system demonstrated the capability for early detection of pathological changes, offering promising applications in noninvasive diagnosis of cancer and other diseases.

**Figure 9:**
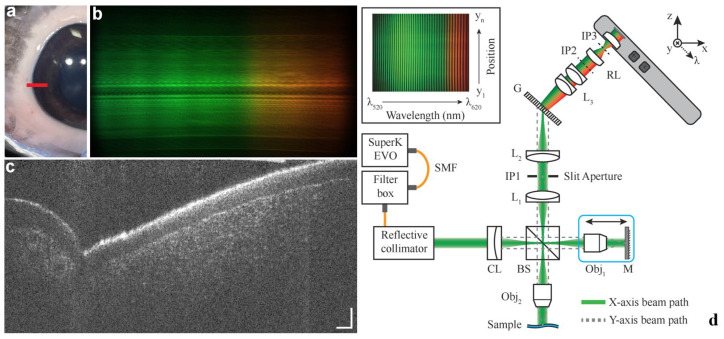
Sample image of ex vivo porcine anterior segment using a smartphone-integrated OCT. Photograph of the anterior segment of the eye (**a**) with the red line showing the location of the B-scan. Raw spectrum (**b**) and 10-frame averaged B-scan (**c**) of the corneal limbus. Scale bars are 150 μm along the y-axis (horizonal) and 50 μm along the z-axis (vertical). (**d**) Optical setup of the smartphone-integrated system. Reproduced with permission from [149] © Optical Society of America.

Miniaturizing vis-OCT probes for minimally invasive high-resolution imaging remains challenging due to severe chromatic aberration and fabrication limitations [139]. Xu et al. recently developed a 0.4 mm diameter ultrathin vis-OCT endomicroscope featuring visible-spectrum optics and customized microlens designs, achieving μm axial resolution in live mouse brains. This system successfully resolved subcellular neural architectures, including nerve fiber bundles in the corpus callosum, neuronal somata, and myelinated axons in the isocortex (Figure 9), with superior boundary sharpness and contrast-to-noise ratio compared to conventional NIR-OCT [140].

### 3.3. Multi-Modality Imaging with Vis-OCT

While vis-OCT offers unique advantages, it also has inherent limitations, such as restricted penetration depth and a lack of molecular specificity beyond hemoglobin absorption. Integrating vis-OCT with other imaging modalities that provide complementary information can overcome these limitations and enable a more comprehensive assessment of tissue structure and function. These multimodal approaches aim to synergistically combine the strengths of different techniques.

Many biologically relevant fluorophores fall within the visible light range, and the integration of vis-OCT with various fluorescence imaging techniques offers a powerful approach to combine high-resolution structural imaging with molecular and cellular specificity. Dai et al. [141] demonstrated a dual-modal system that enables simultaneous acquisition of SD-OCT and autofluorescence microscopy using a shared broadband visible-light source. Since both modalities share the same light source, the resulting images are intrinsically co-registered. Specific autofluorescence signals such as lipofuscin can be quantified by vis-OCT fluorescence systems [142,143,144]. In addition to autofluorescence imaging, exogenous fluorophores have also been used. Most notably, transgenic mice expressing enhanced green fluorescent protein (EGFP) have been widely employed to visualize specific cell populations, such as retinal ganglion cells and microglia [145,146]. These studies demonstrate the capacity of multimodal vis-OCT systems to simultaneously capture structural and molecular information, which is particularly advantageous in neurovascular research where both cellular identity and tissue morphology are crucial.

Vis-OCT can also be integrated with SLO and adaptive optics (AO) to improve its performance in human and animal retinal imaging. In vivo imaging of the human retina using vis-OCT requires precise beam targeting and focus adjustment, which can be particularly challenging due to involuntary eye movements and the limited field of view typical for high-resolution OCT systems. The incorporation of SLO provides a wider-field, almost motion free real-time fundus image, which not only improves the usability of vis-OCT in clinical applications, but it also facilitates its integration with other imaging modalities such as fluorescence imaging. Multimodal combinations of vis-OCT with AO and fluorescence imaging have also been actively explored in preclinical studies. For example, Ju et al. [147] developed a sensorless adaptive optics (SAO)-based multimodal system that combines vis-OCT and fluorescence imaging for high-resolution retinal imaging in mice. The system employs a broadband SC light source to support both modalities, and it applies image-based AO correction using a deformable mirror and an optimization algorithm without requiring a wavefront sensor. By switching between OCT (560 nm) and fluorescence excitation (470 nm) using the same optical path, the system achieves naturally co-registered images of retinal structures and fluorescently labeled features.

### 3.4. Other Functional Extensions of vis-OCT

Functional extensions of NIR-OCT, including polarization-sensitive OCT, have been translated into the visible light regime, where the shorter wavelength improves sensitivity to tissue birefringence. Baradaran et al. [148] recently developed a polarization-sensitive vis-OCT system using a custom bulit spectrometer with single-shot balanced detection using a single line-scan camera, achieving up to 6 dB SNR improvement. Despite limited imaging depth due to increased scattering, the system offered enhanced birefringence contrast and axial resolution of 2.1 μm that allowed clear visualization of tissue microstructure, showing promise for applications in ophthalmology, dermatology, and material science.

Another interesting approach is smartphone-integrated OCT proposed by Malone et al. [149]. A smartphone was used to replace the spectrometer in the vis-OCT system, providing both signal acquisition and image post-processing capabilities. Raw data were captured and processed directly on the smartphone, allowing for enhanced image quality through noise reduction and contrast optimization, as shown in Figure 9. Such integration of consumer-grade technology with OCT provides a novel pathway toward the miniaturization and cost reduction of vis-OCT systems.

### 3.5. Image Processing and Artificial Intelligence Methods for Vis-OCT

Although prior efforts have addressed vis-OCT limitations such as reduced penetration and chromatic dispersion through hardware corrective strategies, residual image degradation remains a critical bottleneck. Issues such as signal dropout, speckle noise, vessel shadowing, and fine-layer ambiguity continue to impair image interpretability and quantitative accuracy. To overcome these limitations, advanced image processing, and AI-based techniques are increasingly employed to denoise, enhance contrast, correct artifacts, and enable reliable feature extraction.

AI-based denoising approaches have been widely explored to address the low SNR limitation. In the context of vis-OCT, special scan modulation methods and DL models, particularly self-supervised methods like Noise2Noise (N2N), Noise2Void (N2V) [150], and Sub2Full (S2F) [151], were used to reduce noise without requiring ground truth. Rubinoff et al. [121] reduced speckle noise by orthogonally modulating B-scans to generate uncorrelated speckle patterns for averaging, which enhanced contrast and preserved fine structural details in both human and mouse retinal imaging. Ye et al. [150] proposed a co-learning deep learning framework that jointly performed self-denoising and retinal layer segmentation. This method enhanced the segmentation accuracy of critical retinal layers, including GCL, IPL, and INL. Compared to models that focus solely on segmentation, the co-learning approach improved the Dice coefficient by approximately 2%. Further experiments demonstrated that the framework retained stable segmentation performance even when trained with only 25% of the annotated data. Wang et al. [151] proposed a self-supervised denoising strategy named Sub2Full, which used two repeated B-scans with split-spectrum and full-spectrum configurations to generate low- and high-resolution vis-OCT images, enabling the network to learn both despeckling and resolution enhancement without clean data. The method outperformed existing self-supervised approaches in reducing speckle noise while preserving fine retinal structures in single B-scan images such as sublaminar layers. These methods improve SNR, contrast, and visualization of fine structures.

Another problem that can be addressed by AI is the prominent vascular shadow artifacts caused by strong hemoglobin absorption at visible wavelengths. These shadows attenuate signals beneath blood vessels and introduce discontinuities in retinal layers, significantly hindering accurate segmentation and quantitative analysis. Ganjee et al. [152] designed a multi-scale Transformer-based segmentation model BreakNet to improve retinal layer segmentation in vis-OCT images by addressing discontinuities caused by vascular shadowing. The network combined global and local feature extraction to mimic expert segmentation strategies, achieving superior performance over U-Net and tightly combined cross-convolution and Transformer with boundary regression and feature polarization (TCCT-BP) while demonstrating strong generalization and robustness to low-quality annotations across different vis-OCT datasets and robustness to low-quality annotations. These artifacts similarly affect image formation in vis-OCTA. Guo et al. [153] developed an end-to-end deep learning approach that directly segments vessels in SVP, ICP, and DCP from volumetric OCTA data. The method achieved the highest F1-score on SVP and demonstrated robust vascular density quantification in the lower-signal ICP and DCP.

It is worth noting that numerous AI-related studies have been conducted across the broader OCT field. This review selectively focuses on methods specifically related to vis-OCT, though many techniques developed for other OCT modalities can in principle be applicable to vis-OCT.

## 4. Discussion

As a branch of OCT technology, vis-OCT utilizes visible light wavelengths to significantly enhance imaging resolution, while the higher scattering coefficient of biological tissues effectively improves image contrast. The strong optical absorption of hemoglobin within the visible spectrum makes vis-OCT particularly suitable for quantitative blood oxygenation measurements. When combined with DOCT for blood flow assessment, vis-OCT serves as a powerful tool for characterizing microvascular system, especially for the retina.

However, compared to NIR-OCT, several challenges hinder the widespread adoption of vis-OCT. One of the most important issues is laser safety. Vis-OCT systems operate at shorter wavelengths than traditional NIR-OCT, bringing incident light power closer to the retina’s photochemical damage threshold. Retinal light exposure can result in injury through the following three primary mechanisms: photothermal, photoacoustic, and photochemical damage, with the last being of greatest concern in the visible range [154]. To mitigate this risk, acquisition parameters must conform to the maximum permissible exposure (MPE) standards outlined in ANSI Z136.1-2014. The MPE is dependent on several factors including exposure duration, beam size, and whether the beam is scanning or stationary. The MPE is approximately 150 µW under the conservative assumption of a stationary exposure for 0.5 s [29]. However, in a scanning OCT system, where the beam moves across a larger retinal area, the permissible power is significantly higher. For example, a 10 s raster scan covering 25 mm${\,}^{2}$ allows a MPE of 7.07 mW [29]. In most human vis-OCT studies, the incident power at the pupil is carefully controlled and typically kept well below the MPE. Many systems operate in the range of 150–250 μW during data acquisition [23,122,123], with lower power used for beam alignment [120]. These protocols ensure safety while maintaining image quality. Compounding this challenge is the high sensitivity of the human eye to visible light, which can generate discomfort and trigger involuntary eye movements during clinical imaging, while reducing illumination intensity can alleviate visual discomfort, it further worsens the already limited sensitivity. Moreover, the higher scattering coefficient of visible light, while improving imaging contrast, simultaneously reduces the imaging depth and sensitivity of vis-OCT. Several balanced detection based vis-OCT approaches have been proposed to mitigate RIN-induced SNR degradation. However, they introduce additional system complexity, leading to higher implementation costs and increased maintenance difficulty.

Another significant limitation of vis-OCT is its scanning rate. The imaging speed of vis-OCT is constrained by the line-scan rate of spectrometer camera, with most studies operating at A-line rates in the tens of kHz range, which is far slower than the MHz-level scanning speeds achievable by SS-OCT systems. As a result, vis-OCT requires several times, or even tens of times, longer acquisition time to complete scans of equivalent field obtained by SS-OCT. Employing faster line-scan cameras could help alleviate this limitation, but further advances in commercial camera technology are still needed. Another possible solution is swept source-based vis-OCT. Apart from achieving higher imaging speeds, SS-OCT also offers superior sensitivity roll-off performance and greater imaging depth compared to SD-OCT. An experimental study [16] have demonstrated imaging speeds up to 100 kHz, along with several-fold improvements in sensitivity roll-off and imaging depth relative to SD configurations, as previously discussed. However, before widespread adoption, challenges related to insufficient optical power and bandwidth still need to be addressed. Moreover, the time-stretch strategy may offer a solution by trading optical spectral resolution for acquisition speed [155], though it also faces challenges such as limited sensitivity and high system cost.

In recent years, vis-OCT has emerged as an increasingly active research field within the OCT community. As vis-OCT technology continues to mature, it can be expected that preclinical studies using animal models will expand, eventually paving the way for widespread clinical applications in ophthalmology. However, the large-scale adoption of vis-OCT faces critical challenge such as the high system cost of SC sources. Alternative configurations, such as low-cost broadband SLD-based sources or SS-vis-OCT systems, may help to mitigate this issue. With ongoing advancements in visible-light communication technologies, breakthroughs in light source development may soon be within reach. Additionally, in practice, clinical observations suggest that sudden changes in light exposure may transiently alter retinal blood flow and metabolic activity [156]. Thus, it is critical to allow patients to adapt gradually to ambient lighting conditions to avoid inducing abrupt physiological responses. Moreover, handheld sample arms may also be needed for populations who are not well-suited to desktop systems, such as children and individuals with disabilities. To enable effective imaging using handheld probes, the development and integration of robust motion compensation and stabilization algorithms will be essential. In addition to outpatient applications, a promising potential use of vis-OCT lies in intraoperative ophthalmic navigation [157]. The clinical value of intraoperative OCT has already been demonstrated with commercially available systems [158,159]. However, advancing the use of vis-OCT in surgical settings requires the development of vis-OCT microscope systems with high imaging speed, wide field of view, high resolution, and extended depth of field, as well as the creation of precise intraoperative navigation algorithms based on vis-OCT imaging.

To drive the large-scale clinical adoption of vis-OCT, another critical aspect is the integration of effective AI methods. AI has already been widely adopted in OCT imaging for tasks such as automated segmentation, disease diagnosis, image quality enhancement, and motion artifact correction. Leveraging AI in vis-OCT can further facilitate automated structural and functional analysis, improve diagnostic accuracy, and compensate for image quality degradation caused by motion or low SNRs, thereby greatly enhancing its clinical feasibility and efficiency.

Vis-OCT applications in other anatomical sites are also emerging as promising directions. Existing studies have extended vis-OCT to brain imaging, skin imaging, and endoscopic application. However, a major limitation hindering broader *in vivo* applications is the relatively shallow penetration depth of visible light. Many pathological targets lie beyond the imaging range of vis-OCT, especially in internal organs. To overcome this barrier, the development of specialized endoscopic tools, such as intravascular catheters and needle-based probes, is essential to deliver and collect light from deeper regions. Alternatively, tissue optical clearing techniques [160] may also be employed to enhance light penetration and improve imaging performance in otherwise inaccessible areas for certain applications.

## 5. Conclusions

Vis-OCT has emerged as a promising imaging modality that offers exceptional spatial resolution and functional capabilities, particularly in retinal imaging and ocular disease assessment. By leveraging the unique optical properties of visible light, vis-OCT enables high-resolution structural imaging and quantitative oximetry, surpassing the performance of conventional NIR-OCT in several aspects.

Despite its advantages, vis-OCT still faces notable technical and practical challenges, including limited imaging depth, restricted ocular safety thresholds, high system cost, and relatively slow imaging speed. Recent developments in balanced detection schemes, swept-source configurations, and AI-assisted post-processing have begun to address these limitations. Furthermore, emerging applications such as brain imaging and endoscopic imaging are broadening the potential clinical impact of vis-OCT beyond ophthalmology.

With continuous advancements in light source technologies, detection systems, and intelligent imaging algorithms, vis-OCT is well positioned to transition from a research-focused tool to a practical and versatile modality for clinical diagnostics across a wide range of biomedical fields.
